# A genetic system for targeted mutations to disrupt and restore genes in the obligate bacterium, *Ehrlichia chaffeensis*

**DOI:** 10.1038/s41598-017-16023-y

**Published:** 2017-11-17

**Authors:** Ying Wang, Lanjing Wei, Huitao Liu, Chuanmin Cheng, Roman R. Ganta

**Affiliations:** 1 Center of Excellence for Vector-Borne Diseases (CEVBD), Department of Diagnostic Medicine/Pathobiology, College of Veterinary Medicine, Kansas State University, Manhattan, KS 66506 USA; 2 Present Address: Vanderbilt Technologies for Advanced Genomics, Vanderbilt University Medical Center, Nashville, TN 37232 USA

## Abstract

Obligate intracellular bacteria (obligates) belonging to Rickettsiales and Chlamydiales cause diseases in hundreds of millions of people worldwide and in many animal species. Lack of an efficient system for targeted mutagenesis in obligates remains a major impediment in understanding microbial pathogenesis. Challenges in creating targeted mutations may be attributed to essential nature of majority of the genes and intracellular replication dependence. Despite success in generating random mutations, a method that works well in creating mutations in specific genes of interest followed by complementation remains problematic for obligates and is a highly sought-after goal. We describe protocols to generate stable targeted mutations by allelic exchange in *Ehrlichia chaffeensis*, an obligate intracellular tick-borne bacterium responsible for human monocytic ehrlichiosis. Targeted mutations in *E. chaffeensis* were created to disrupt two genes, and also to restore one gene by another allelic exchange mutation leading to the restoration of transcription and protein expression from the inactivated gene and the recovered organisms also express mCherry, which distinguishes from the wild type. We expect that the methods developed are broadly applicable to other obligates, particularly to rickettsial pathogens, to routinely perform targeted mutations to enable studies focused on protein structure-function analyses, host-pathogen interactions and in developing vaccines.

## Introduction

Obligate intracellular bacteria are responsible for causing diseases in hundreds of millions of people worldwide. They include many pathogenic Gram-negatives of the orders Rickettsiales and Chlamydiales^1–3^. Lack of an efficient system for targeted mutagenesis in obligates belonging to Rickettsiales and Chlamydiales of the genera *Ehrlichia*, *Anaplasma*, *Rickettsia*, *Orientia* and *Chlamydia* remains a major impediment in understanding microbial pathogenesis and in defining the functional significance of many genes of the obligates. Chlamydiales and Rickettsiales have undergone extreme genome reductions^4–7^ where the majority of genes for each pathogen may be critical for their intracellular growth. Thus, obligates depend on their hosts to fill in the deficiencies resulting from genome reductions. Consistent with this hypothesis, prior studies demonstrate that nearly 74-92% of the predicted genes in *Ehrlichia*, *Anaplasma*, *Rickettsia*, and *Chlamydia* species are transcriptionally active during bacterial replication in the host cells of vertebrates and vectors^8–11^. Challenges in creating targeted mutations may be attributed to the essential nature of a gene selected for mutagenesis, intracellular replication dependence and the lack of methods to support extracellular growth. Despite the success in generating random mutations using transposon mutagenesis^12–17^, and having a limited success of creating targeted mutations in rickettsial pathogens^15,18^, presently a method that works well in creating targeted mutations in specific genes of interest followed by complementation remains problematic for the obligate pathogens and it is also a highly sought-after goal^19–23^. We have filled this major methodological deficiency by developing protocols to generate stable targeted mutations by allelic exchange in *Ehrlichia chaffeensis* where we could disrupt two genes, and also restore the intact gene by another allelic exchange mutation in one of the two genes, resulting the restored transcription of the inactivated gene from its own promoter.

*E*. *chaffeensis* is a tick-transmitted rickettsial bacterium and is the causative agent of human monocytic ehrlichiosis (HME)^7^. HME is an emerging infectious disease in the USA and is also frequently reported from other parts of the world^24^. It is an acute flu-like illness having clinical signs including fever, headache, myalgia, anorexia and chills and is commonly associated with leukopenia, thrombocytopenia, anemia, and upgraded levels of serum hepatic aminotransferases^2^. Similarly, several other *Anaplasmataceae* family pathogens, including the genera *Ehrlichia* and *Anaplasma*, have been identified in recent years as the causative agents of important emerging diseases in people and various vertebrate animals^2,25,26^. The limited availability of genetic tools to study obligate intracellular pathogens, including the genera *Ehrlichia*, *Anaplasma*, *Rickettsia* and *Chlamydia*, particularly in creating targeted mutations restrict our understanding of the molecular mechanisms of pathogenesis and how the pathogens overcome host clearance^27–29^. Recently, we reported both random and targeted mutations in *E*. *chaffeensis* ^15^. Himar1 transposase based random mutagenesis is efficient in creating mutations in both protein coding and non-coding regions of several genes^15^. Himar1 random mutagenesis aided in elucidating host-pathogen interactions and assessing the value of mutants for development of attenuated mutant vaccines^30,31^. We also described targeted mutations at three genomic sites by allelic exchange and by group II intron mutagenesis methods; however, the targeted mutations persisted only for few days in cell cultures^15^. These data suggest that the targeted mutagenesis requires considerable standardization.

In this study, with the primary goal of optimizing methods for disrupting and restoring a gene of interest in obligates, we carried out allelic exchange mutation experiments in two *E*. *chaffeensis* genes, Ech_0230 and Ech_0379, where we previously reported stable insertion mutations by transposon mutagenesis^15^. Mutagenesis constructs were prepared and successfully used in disrupting the two genes and then in restoring one of the two inactivated genes, Ech_0379.

## Results

**Figure 1:**
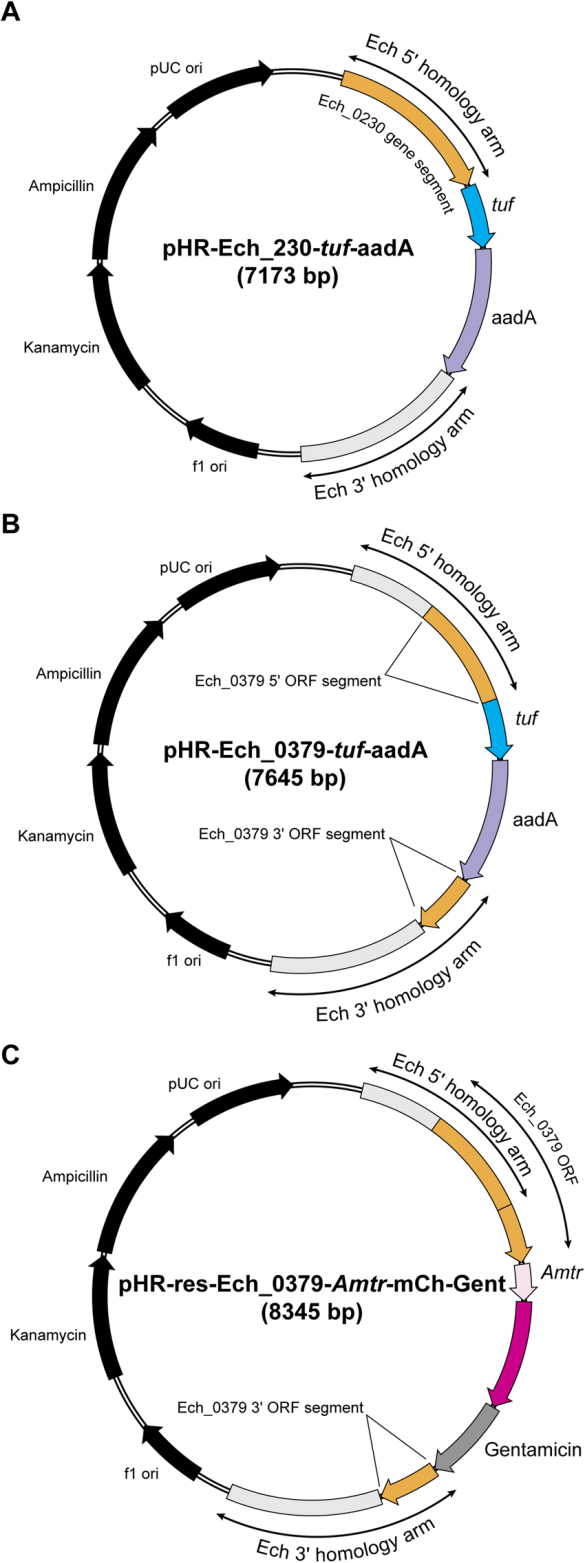
Plasmid maps of pHR-Ech_0230-*tuf*-aadA (**A**), pHR-Ech_0379-*tuf*-aadA (**B**) and pHR-res-Ech_0379-*Amtr*-mCh-Gent (**C**). Homology arms for targeting allelic exchange experiments were identified in all three plasmids. The plasmid sequence data for all three constructs were deposited in the GenBank (accession #s MF068805, MF068806, and MF068807, respectively).

The detailed schematic representation of the strategies employed for creating allelic exchange mutations to disrupt two *E*. *chaffeensis* genes (Ech_0230 and Ech_0379) and to restore one of the disrupted genes Ech_0379 in Supplementary Fig. S1 and the plasmid constructs generated in this study are presented Fig. 1. More details about the development of constructs and their application for targeted mutagenesis are outlined below.

### Construction of homologous recombination plasmids

We previously reported transposon insertion downstream to the coding region of Ech_0230 gene resulting in the loss of gene activity. Similarly, transposon mutation within the coding region of Ech_0379 resulted in the transcriptional inactivation^15^. To generate targeted mutations at the same genomic locations, about 1 kb of *E*. *chaffeensis* genomic DNA segments upstream and downstream of the previously defined mutation insertion sites of the Ech_0230 and Ech_0379 genes were obtained by PCR and cloned into a plasmid vector. The promoter segment of *E*. *chaffeensis* elongation factor Tu gene, *Tuf*-*2*, (Ech_0407) was similarly cloned in front of the aadA gene coding sequence into a separate plasmid (aadA gene confers resistance to spectinomycin and streptomycin)^12^. *Tuf-2* gene promoter (*tuf*) was chosen for aadA gene transcription because it drives the expression of a highly conserved and constitutively expressed protein (Tu) that is necessary for the polypeptide elongation process in the protein translation machinery^32^. Further, our bioinformatics analysis and transcription mapping by primer extension experiment suggested that it is a strong promoter responsible for transcribing 25 genes, most of which encode for 30S and 50S ribosomal proteins, and having multiple transcription start sites (not shown). The aadA gene was chosen as it works well in conferring antibiotic resistance in *E*. *chaffeensis* and in *Anaplasma* species^13,15^. The *tuf*-aadA segment was engineered into the homologous recombination plasmid constructs of Ech_0230 and Ech_0379 (named as pHR-Ech_0230-*tuf*-aadA and pHR-Ech_0379-*tuf*-aadA, respectively) (Fig. 1). Linear DNA fragments from the plasmid constructs containing the 5′ and 3′ homology arms of the genes flanking the *tuf*-aadA segment were generated by PCR for use in creating targeted mutations.

To prepare a rescue mutagenesis template to reverse the intact gene of one of the targeted gene mutations within the Ech_0379 gene, a 0.4 kb fragment downstream from the mutation site of the gene was generated by PCR from *E*. *chaffeensis* genome and it was engineered into the pHR-Ech_0379-*tuf*-aadA construct to create a modified recombinant plasmid; pHR-res-Ech_0379-*Amtr*-mCh-Gent containing the entire Ech_0379 gene ORF followed by the presence of the *Amtr* promoter^12^, the ORFs of mCherry and the gentamicin resistance cassettes (Gent) (*Amtr*-mCh-Gent) and a 1 kb genomic segment containing the 3′ portion of the Ech_0379 gene (Fig. 1). Gent was codon optimized for efficient translation in *E*. *chaffeensis* (Supplementary Fig. S2). Linear fragments from the plasmid were prepared from the construct containing the 5′ homology arm including the Ech_0379 gene ORF followed by mCh-Gent segment driven from *Amtr* promoter and the 3′ end genomic segment downstream to the Ech_0379 insertion to serve as the 3′ homology arm (Fig. 1).

### Transformation of *E*. *chaffeensis* with the linear DNA fragments to promote disruption mutations by allelic exchange

**Figure 2:**
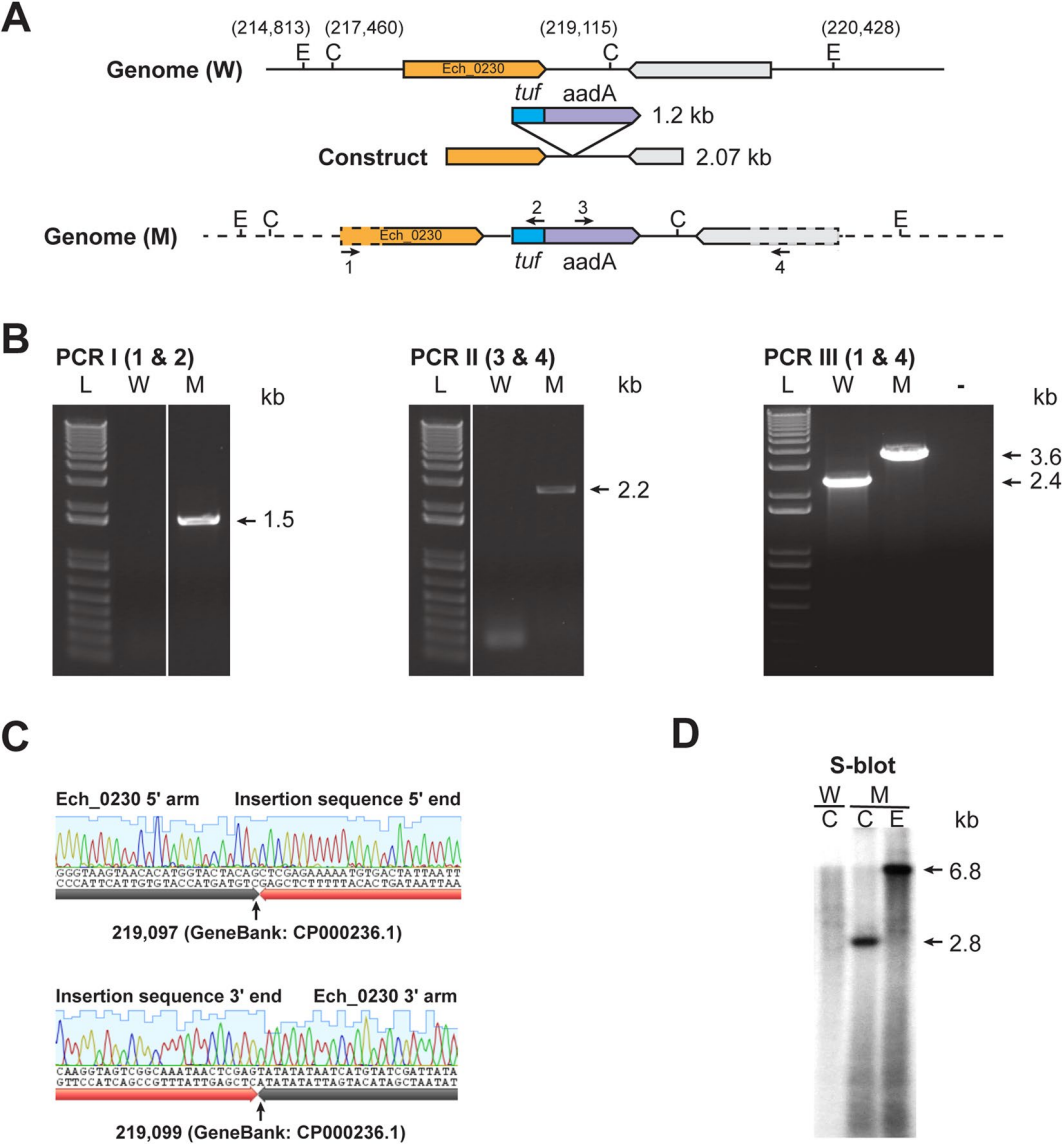
Targeted allelic exchange mutagenesis to disrupt Ech_0230 gene. (**A**) An illustration depicting the genomic segment spanning the region selected for preparing allelic exchange construct, including the restriction enzyme sites {EcoRI (E) and ClaI (C)} used for the mapping the insertion. Genomic coordinates for restriction enzyme sites and the size of inserted fragment (*tuf*-aadA) were included to allow determination of the expected DNA sizes in PCR and Southern blot analysis. (**B**) Amplicons resolved following three different PCRs using primers targeting to the genomic regions upstream and downstream to the allelic insertion (primers identified as 1 and 4) and to the inserted DNA (primers; 2 and 3). (L, 1 kb plus molecular weight DNA markers; W, PCR with wild type genomic DNA as the template; M, PCR with mutant genomic DNA as the template). (**C**) PCR DNA Sequence verification of insertion sites in the targeted mutant. DNA sequence generated from amplicons (panel B); sequence shown above black arrow lines represents the sequence from *E*. *chaffeensis* genome, while the sequence above the orange arrowhead lines represents the inserted sequence in the gene disruption mutant. Sequences boundaries at the 5′ and 3′ insertion junctions were identified with a small black arrow lines. (**D**) Southern blot analysis of genomic DNAs (W and M) digested with ClaI (**C**) or EcoRI (**E**). The blot analysis was performed with aadA gene segment as the probe. (Full-length gels and blots were included in the Supplementary Figure file, as parts of the Figure had cropped images).

**Figure 3:**
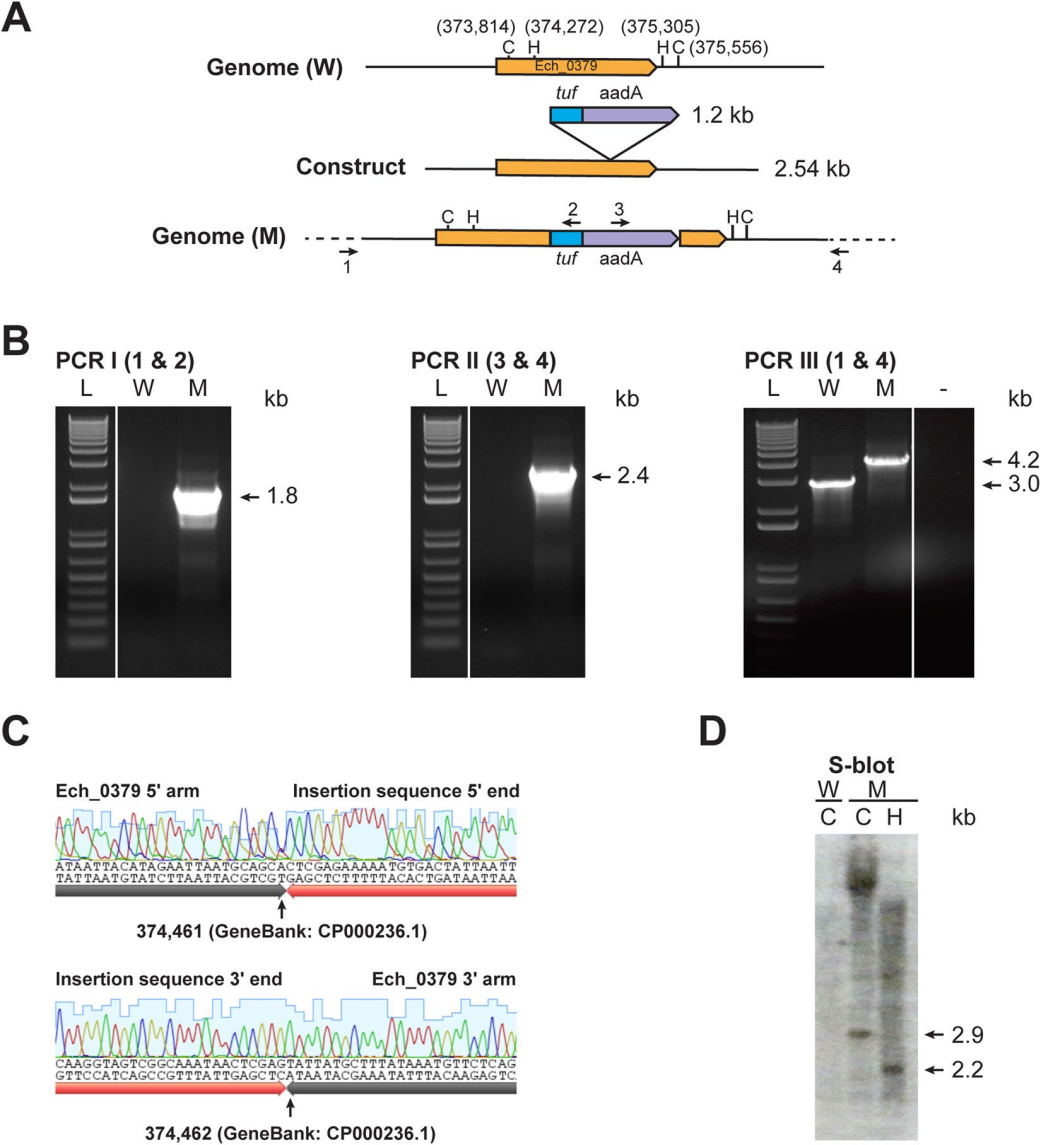
Targeted allelic exchange mutagenesis to disrupt Ech_0379 gene. Panels A, B, C and D are as in Fig. 2, except that the restriction enzymes ClaI (C) and HindIII (H) were used for genomic mapping (**A**) and for Southern blot analysis (**D**). (Full-length gels and blots were included in the Supplementary Figure file, as parts of the Figure had cropped images).

Linear recombinant DNA fragments to disrupt Ech_0230 or Ech_0379 genes (Figs 2A and 3A) were electroporated into host cell-free wild type *E*. *chaffeensis* organisms recovered from ISE6 tick cells. The electroporated cells were transferred to ISE6 tick cell suspension and propagated in the absence of antibiotics for 24 hours at 34 °C at which point the media was supplemented with spectinomycin and streptomycin for selecting pure targeted gene disruption mutant organisms. The mutants were selected for their ability to grow in the medium containing the antibiotics for several weeks. Cell-free mutant dense core organisms were collected from ISE6 culture and inoculated into DH82 cultures for continuous growing in this macrophage cell line as well. Transformants resisting to the presence of antibiotics in the media were observed typically in about two weeks and remained in continuous cultures of tick cells and also in macrophage cultures for several months.

### Confirming the presence of *E*. *chaffeensis* mutants by PCR, DNA sequence verification and Southern blot analysis

Following the recovery of *E*. *chaffeensis* cultures growing in the media containing antibiotics, targeted gene disruption in Ech_0230 or Ech_0379 was first detected by two insertion specific PCR assays targeting 1) to the genomic region 5′ to the allelic exchange site and to the insertion specific DNA (PCR I), 2) to the insertion DNA and to the 3′ of the allelic exchange site on the genome (PCR II). Clonal purity was then confirmed by another PCR assay (PCR III) targeting the genomic regions upstream and downstream of the allelic exchange insertion sites (Figs 2B and 3B). In particular, we did not detect the smaller amplicon that is expected for the wild type *E*. *chaffeensis* in PCR III, thus validating that the organisms are clonally pure. The integrity of the PCR products was confirmed by PCR-DNA sequence analysis (Figs 2C and 3C). The presence of insertion mutations and clonal purity of the targeted mutations was further confirmed in the ClaI and HindIII restriction enzyme digested genomic DNAs by Southern blot analysis using the aadA gene probe; predicted restriction enzyme digested DNA fragments were detected only in the mutant cultures, but not in the DNA from wild type *E*. *chaffeensis* (Figs 2D and 3D).

### Transformation of *E*. *chaffeensis* Ech_0379 mutant with the linear DNA fragments to promote restoration mutation by allelic exchange

**Figure 4:**
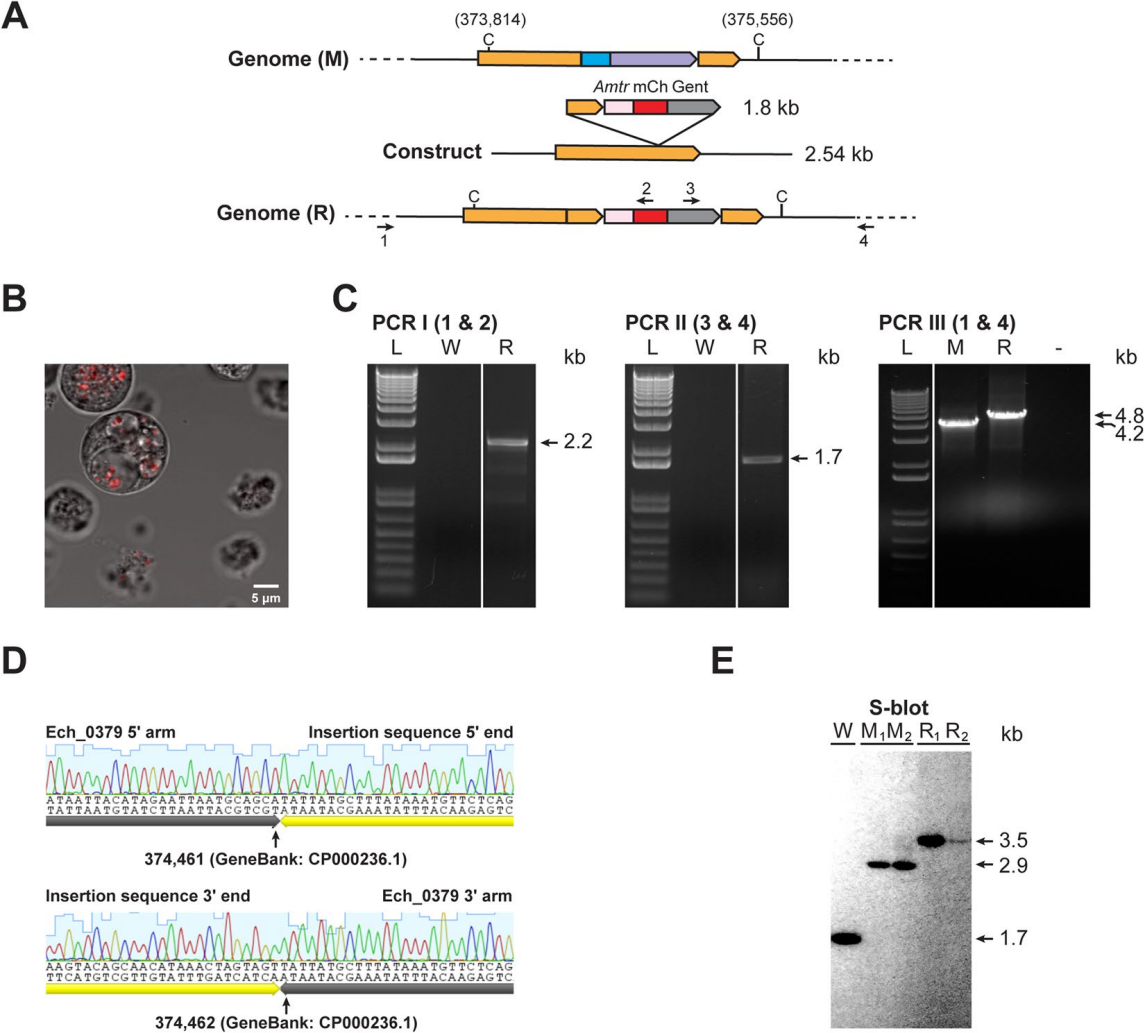
Targeted allelic exchange mutagenesis to restore Ech_0379 gene. Panel A is similar to Fig. 2 except that the illustration depicting the genomic segment at the top portion of the panel represents the genome from Ech_0379 mutant. (**B**) Ech_0379 gene restoration mutant culture expressing mCherry. The restored mutant organisms cultured in ISE6 cells were assessed for the mCherry expression by confocal microscopy using 40x magnification lens. Panels C, D, and E are similar to Fig. 2 panels B, C and D, respectively, except that the inserted sequence in the gene disruption mutant is identified in yellow arrowhead line (panel D) and restriction enzyme and probe used for the Southern blot experiment (panel E) were Cla I and a DNA segment representing Ech_0379 gene, respectively. In panel E, the lanes M_1_ and M_2_ represent data from genomic DNA recovered from the *E*. *chaffeensis* Ech_0379 mutant culture recovered from DH82 and ISE6 culture, respectively. Similarly, R_1_ and R_2_ represent data from genomic DNA recovered from the *E*. *chaffeensis* Ech_0379 reverted mutant culture recovered from DH82 and ISE6 culture, respectively. (Full-length gels and blots were included in the Supplementary Figure file, as parts of the Figure had cropped images).

For rescue mutation experiment, linear DNA fragments of the Ech_0379 gene restoration template (Fig. 4A) were similarly electroporated into *E*. *chaffeensis* organisms containing mutation in the Ech_0379 gene. *E*. *chaffeensis* cultures with Ech_0379 gene restored were then selected by their ability to grow in the medium containing gentamicin. Further, the gene restoration mutant was assessed for the mCherry protein expression by fluorescence microscopy (Fig. 4B). Subsequently, we verified the presence of restoration mutation and clonal purity by three different PCRs targeting to 5′ or 3′ to the allelic exchange regions and to the inserted antibiotic resistance segment (PCR I and II) and using primers targeted outside of the allelic exchange sites (PCR III); expected size amplicons were observed in all three PCR experiments (Fig. 4C) and the sequence integrity was then confirmed by PCR DNA sequence analysis (Fig. 4D). The PCR III also confirmed that the rescue mutant is clonally pure. Southern blot analysis using a Ech_0379 gene segment as the probe further confirmed the presence of the clonally pure restoration mutation, as the expected larger DNA fragments were only observed for the ClaI digested genomic DNA, compared to those detected for DNA from wild type and Ech_0379 gene disruption mutant organism (Fig. 4E). Subsequent to establishing the clonal purity, cultures of the mutants were maintained continuously in the absence of added antibiotics to the culture media.

### Targeted gene knockout and restoration mutations verified by RT-PCR

**Figure 5:**
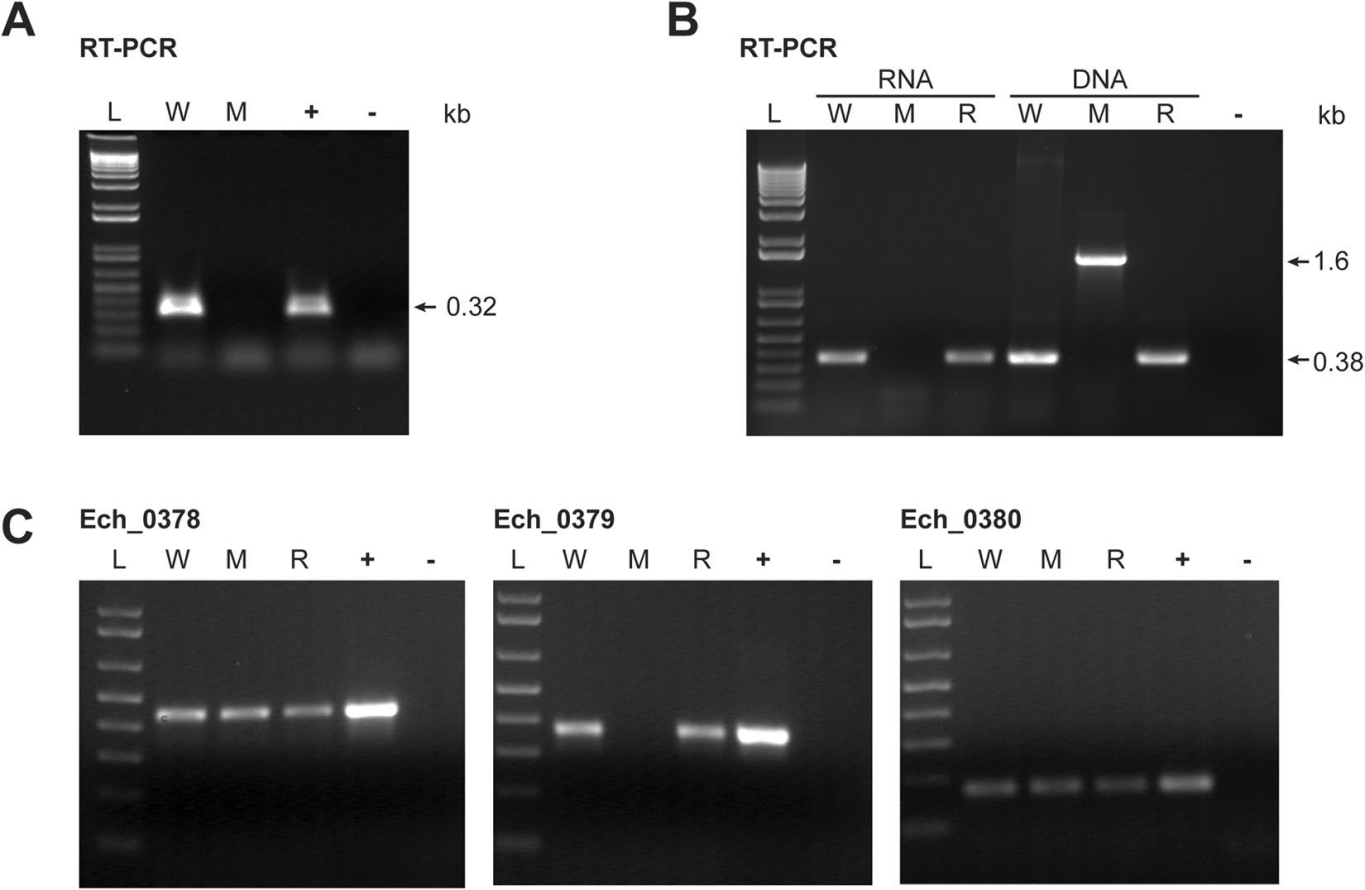
Transcriptional analysis of RNA recovered from wild type and allelic exchange mutant *E*. *chaffeensis* organisms assessed by RT-PCR. (**A**) RT-PCR products from wild type (W) and Ech_0230 mutant (M) organisms were resolved (L, 1 kb plus molecular weight DNA markers resolved; +, genomic DNA from wild type *E*. *chaffeensis* was used as the template; -, negative control reaction with no template added). (**B**) As in panel A, except that the analysis was performed using RNA recovered from Ech_0379 disruption (M) and restoration (R) mutant organisms. Positive controls for this experiments included genomic DNAs as the templates from W, M and R. (0.38 kb amplicons are expected for DNA templates in PCRs of W and R and 1.6 kb product is expected for M DNA as the template.) (**C**) Mutations to inactivate and restore the gene activity in Ech_0379 did not alter the gene expression from its neighboring genes. Semi-quantitative RT-PCR assays were performed at 30, 35 and 40 PCR cycles for Ech_0378, Ech_0379 and Ech_0380 for wild type, gene inactivation and gene rescue mutant organisms and the data for 35 cycles were presented. W, M and R had similar quantities of amplicons for Ech_0378 and Ech_0380; Ech_0379 amplicons were also similar for W and R, while absent for M. (Full-length gels and blots were included in the Supplementary Figure file, as parts of the Figure had cropped images).

RT-PCR analysis revealed that the Ech_0230 and Ech_0379 transcripts were present in wild type *E*. *chaffeensis* and were absent in the gene disruption mutant organisms (Fig. 5A,B). The restoration mutant strain tested positive for the Ech_0379 transcript similar to wild type *E*. *chaffeensis* (Fig. 5B). Further, we tested if the allelic exchange mutations to inactivate and restore gene activity in Ech_0379 may have caused polar effects in altering the gene expression from its neighboring genes. The analysis was carried out by semi-quantitative RT-PCR assays where three sets of PCR cycles were used; 30, 35 and 40. Independent of the numbers of PCR cycles performed, RT-PCR products were similar for Ech_0378 and Ech_0380 for wild type, gene inactivation mutant and gene rescue mutant, and Ech_0379 RT-PCR products were absent only in the gene inactivation mutant, while appeared similar for wild type and gene rescue mutant (Fig. 5C).

### Phenotypic characterization of Ech_0379 gene product from wild type, gene disrupted and gene restored *E*. *chaffeensis*

**Figure 6:**
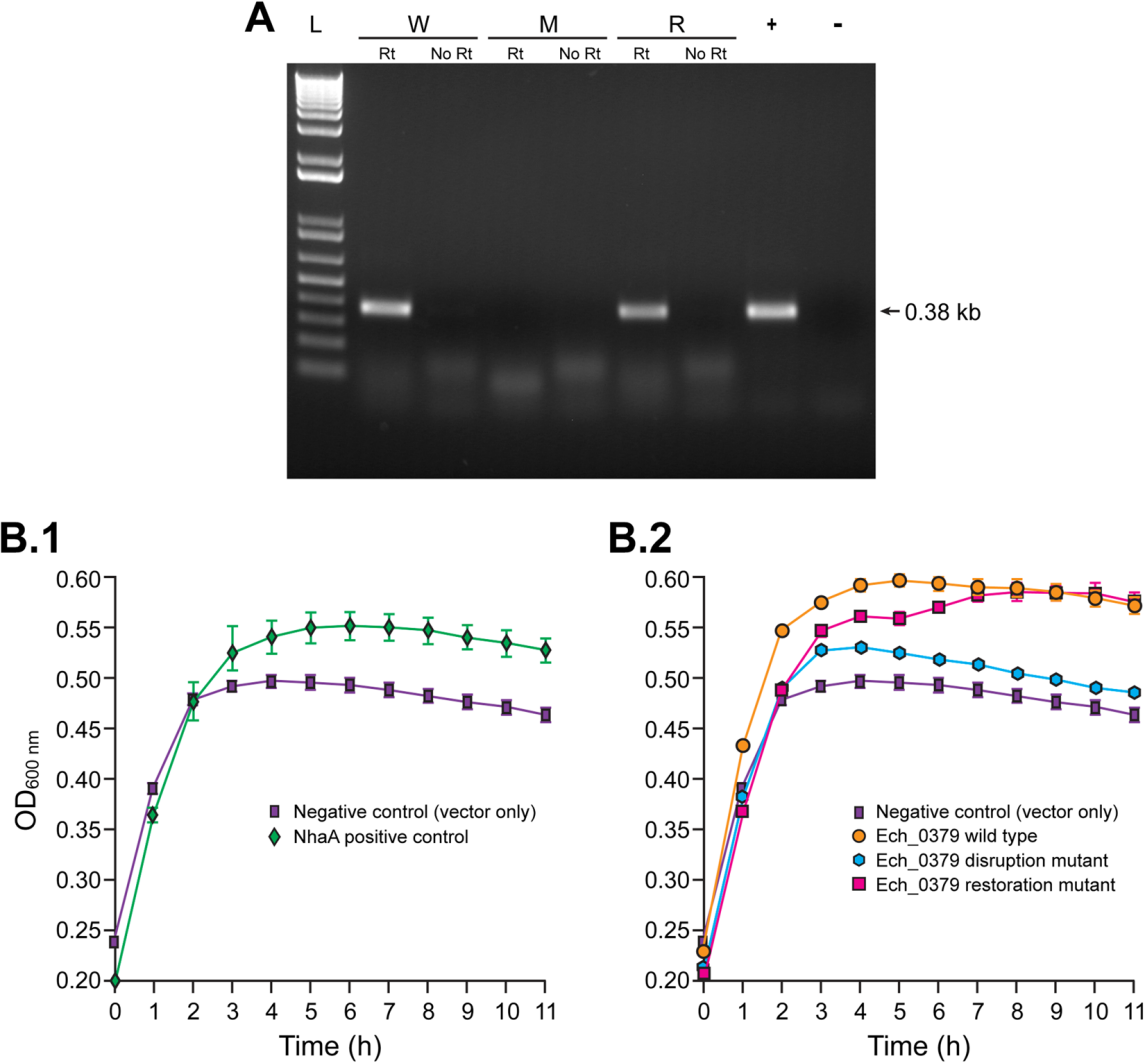
Phenotypic characterization of Ech_0379 gene in antiporter deficient *E*. *coli* strain EP432. (**A**) RT-PCR analysis targeting to Ech_0379 transcripts in EP432. EP432 strain of *E*. *coli* transformed with recombinant plasmids containing the Ech_0379 gene from wild-type (W), gene disruption (M) and restoration (R) mutant organisms were assessed. (L, 1 kb plus molecular weight DNA markers; Rt, with reverse transcriptase; No Rt, without reverse transcriptase; +, wild type genomic DNA used as the template (positive control); −, negative control with no template added). (**B**) The growth of EP432 strain having different recombinant plasmids in LB media containing 200 mM NaCl. The bacterial growth was measured as OD at 600 nm. Three independent experiments were done and the average data were presented with bars showing standard deviation.

We previously predicted that the Ech_0379 gene encodes for an antiporter gene^15^. *E*. *coli* antiporter gene mutant strain, EP432 having mutations in two of the three antiporter genes^33^, is exploited as a great research tool in studying antiporter proteins of several Gram negative bacteria^34,35^. In particular, this *E*. *coli* strain is used in mapping the functions of antiporter proteins by functional complementation assays. Complementation assays are performed in conferring resistance to its Na^+^ sensitivity in high NaCl concentrations in a growth medium. To define the function of Ech_0379 gene product in *E*. *chaffeensis* and also to assess the impact of targeted disruption and complementation mutations, we adopted the *E*. *coli* complementation assay using the EP432 strain. We cloned the Ech_0379 gene sequences, including its native promoter, from wild-type, gene disruption or gene restoration mutant organisms into a plasmid and then transformed the plasmids individually into the EP432 strain. To serve as a positive control, *E*. *coli* NhaA (one of its missing antiporter genes) is similarly cloned and transformed, while non-recombinant plasmid transformed culture of the strain was used as a negative control. DNA-free total RNAs recovered from *E*. *coli* strains containing the Ech_0379 genes were assessed for the presence of the gene transcripts by RT-PCR (Fig. 6A). Predicted amplicons were detected only for RNAs recovered from the *E*. *coli* containing Ech_0379 gene from the wild-type and the gene restoration mutant of *E*. *chaffeensis*, but not in the RNA from the strain containing the disruption mutant *E*. *chaffeensis* gene. We then tested the *E*. *coli* cultures for the antiporter protein activity by functional complementation assay to rescue its Na^+^ sensitivity (Fig. 6B). The growth of the *E*. *coli* in media containing 200 mM NaCl was greater for the strain transformed with the plasmid containing the NhaA gene (positive control), compared to the strain containing a non-recombinant plasmid (negative control) (Fig. 6B.1). The EP432 growth at 10 and 11 h time points were compared by student’s *t*-test; significant differences were observed between the negative and positive controls (p < 0.005). Similarly, the *E*. *coli* containing the Ech_0379 gene from wild type and restoration mutant *E*. *chaffeensis* had enhanced growth compared to the negative control (Fig. 6B.2). The measurements were not significantly different for these two genes (p > 0.1), while the bacterial growth for the disruption mutant gene for 10 and 11 h of assessments were significantly different compared to wild type or restoration mutant gene containing *E*. *coli* strains (p < 0.0001). The growth of the *E*. *coli* having the Ech_0379 gene from disruption mutant *E*. *chaffeensis* was very similar to the negative control.

Successful development of targeted mutations by homologous recombination, which remained continuously in culture of both tick cells and macrophage cell line for several months of assessment, was observed for the two genes of *E*. *chaffeensis* selected in the current study (Ech_0230 and Ech_0379). Complementation mutation to restore the gene activity from one of the two genes (Ech_0379) was also accomplished when targeted to the mutated region. The presence of insertions in the *E*. *chaffeensis* genome was confirmed by the development of antibiotic resistance, using three independent molecular methods; PCR, PCR DNA sequence and Southern blot analysis. Further, the loss of gene function and the restoration were verified by RT-PCR and by protein functional complementation assay in the *E*. *coli* strain having an antiporter gene mutation.

## Discussion

This study included the detailed description of methods in generating stable mutations by homologous recombination in an obligate bacterium where we report the disruption of two genes resulting in the transcriptional silencing and then restoration of the integrity of one of the two genes. All three mutations grew continuously in both ISE6 and DH82 host cells for several months of assessment. There was no evidence to support the presence of off-target insertions developed during all the three mutational experiments. Moreover, the restoration mutant is similar to wild type and yet it can be discriminated from wild type, as it expresses the fluorescent protein, mCherry. In the restoration mutant, open reading frame of the inactivated gene is completely restored in front of its own promoter. Its gene structure is, therefore, similar to the wild type *E*. *chaffeensis*, except that it also expresses mCherry and gentamicin resistance proteins. This modified *E*. *chaffeensis*, that is similar to wild type, will be useful for studies in monitoring the pathogen in real time by fluorescence imaging *in vitro* and *in vivo*, similar to prior studies described for *Borrelia burgdorferi* ^36^.

In this study, we carefully selected promoters, antibiotic resistance genes and cell lines in improving the efficiency of targeted mutations. Based on previous targeted and random mutagenesis experiments of Anaplasmataceae family pathogens belonging to the genera *Ehrlichia* and *Anaplasma*, a greater mutagenesis success is reported with the tick cell line, ISE6^12,15,16^. Therefore to maximize our efforts in succeeding the mutants’ generation, we routinely performed mutagenesis experiments in ISE6 cells. We believe that the use of *tuf* promoter and the development of a codon-optimized Gent are broadly applicable for mutagenesis experiments in other obligate bacteria, particularly for the rickettsial pathogens. Additional minor modifications, such as exchanging a *tuf* promoter and further codon optimization of antibiotic resistance cassettes, may be required in enhancing the mutation success when working with other distantly related obligate bacteria. The *Amtr* promoter, aadA and mCherry ORFs are frequently and successfully used in mutagenesis experiments in Rickettsiales^12,13,15^ and our current study validated their broader applicability.

Ability to create targeted mutations to disrupt and also to restore the integrity of a gene is of exceptional significance to the studies of obligate bacteria; our study represents a major advancement for the research in defining the functions of many proteins likely contributing to pathogenesis and in altering a host response, as these methods are not fully developed up to now and also is a highly sought after goal, most certainly for rickettsiales and chlamydiales^22,23^. Recently, targeted disruption and restoration of a gene is described for *R*. *rickettsii* using the group II intron based mutagenesis method^21^. While this is encouraging, group II intron-based mutagenesis is not broadly applicable for many gene targets, as the identification of optimal sequences within a gene by the proprietary algorithm is necessary prior to any attempts in creating disruption and restoration mutations. Similarly, a recent study described the application of a suicide vector to create targeted mutations in *Chlamydia trachomatis* as a way of developing genetic manipulation by allelic exchange and the method introduced a fluorescent protein to allow monitoring of mutagenesis by fluorescence microscopy^20^. Likewise, our study also included the expression of a fluorescent protein to permit monitoring the bacterial growth, a significant benefit when manipulating intracellular bacteria. Moreover, we are the first to demonstrate a complementation method for an obligate using a homologous recombination method.

Currently, transposon mutagenesis to create random mutations is the only major alternative for investigations on obligates focused on identifying genes essential for understanding pathogenesis and host-pathogen interactions, including efforts in developing attenuated vaccines^12,15,16,30,31,37,38^. However, transposon mutagenesis method cannot be used in targeting a gene of interest. Targeted mutagenesis methods, described in the current study, are broadly applicable in creating similar mutations in other related pathogenic rickettsiales to test molecular Koch’s postulates to define a specific gene function^19,39^.

Ability to create allelic exchange mutations has many applications; for example, it can be used in inserting a DNA to express antisense RNA to knockdown the expression from a gene of interest or to define the function of a hypothetical gene. Additionally, protein tags, such as fluorescent proteins or Histidine tags, could be engineered as bacterial fusion proteins to serve as translational markers^40^ useful in defining protein-protein interactions, and to map secretions of bacterial effectors into host cell cytoplasm, mitochondria and/or nucleus^41–44^. Despite the identification of several bacterial secretory effectors by various secretory systems as influencing the host responses^45^, much remains to be understood in identifying and defining the functions of many unknown effector proteins. Two distinct antibiotic resistance cassettes and promoter segments described in this study for creating allelic exchange mutations will be valuable in generating dual mutants to study interactions of two or more gene products. Targeted disruption to study an essential gene of an obligate may require innovative gene manipulation strategies, such as disrupting only parts of the functional domains of a protein critical for the bacterial growth. The methods in creating allelic exchange mutations reported in this study, therefore, are expected to serve as a catalyst in initiating detailed reverse genetic studies in defining microbial pathogenesis, bacterial and host protein-protein interactions, immunity and vaccine development in several obligates impacting human and animal health.

## Materials and Methods

### *In vitro* cultivation of *E. chaffeensis*

*E*. *chaffeensis* Arkansas isolate was continuously cultivated in the *Ixodes scapularis* embryonic cell line (ISE6) and in the canine macrophage cell line (DH82) as described earlier^46^.

### Construction for homologous recombination plasmids and segments

All primers used for preparing recombinant plasmid constructs for the targeted mutagenesis experiments are described in Supplementary Table S1. Plasmids described in this study were listed in Supplementary Table S2. The Platinum® Taq DNA Polymerase High Fidelity (Invitrogen, Carlsbad, CA) was used in all PCR experiments for preparing the constructs. About 2.0 kb genomic DNA segments spanning about 1 kb each from both sides of the previously identified mutation insertion sites of the Ech_0230 and Ech_0379 genes were generated by PCR using *E*. *chaffeensis* genomic DNA as the template. Genome coordinates (GenBank # CP000236.1) of the amplified segments of Ech_0230 and Ech_0379 genes are 218,060 to 220,133 and 373,265 to 375,810, respectively. The amplicons were cloned into the pCR™2.1-TOPO TA vector (Life Technologies, Rockville, MD). Linear fragments were then generated from the entire plasmids (pHR-Ech_0230 and pHR-Ech_0379 respectively) containing the gene segments using Ech_0230 or Ech_0379 gene specific primers designed to split these gene fragments to two equal halves positioned at each end of the linear fragments and keeping the plasmid backbone in the middle (fragment 1). *Tuf*-*2* gene (Ech_0407) promoter (*tuf*) spanning 0.37 kb DNA was generated by PCR using *E*. *chaffeensis* genomic DNA as the template (genome coordinates are 396,385 to 396,751). The aadA gene open reading frame (ORF) was obtained by PCR from pCis mCherry-SS Himar A7 plasmid^15^. The *tuf* promoter and aadA ORF were also cloned into a separate pCR™2.1-TOPO TA vector and used to generate linear fragments of *tuf-*aadA segment (fragment 2). By following the protocols of Gibson Assembly method (New England Biolabs, Ipswich, MA), the linear fragments 1 and 2 were assembled to create the final homologous recombinant plasmid constructs, where the gene segments were disrupted with the insertion of *tuf-*aadA segment. The final constructs were named as pHR-Ech_0230-*tuf-*aadA and pHR-Ech_0379-*tuf-*aadA, respectively (Fig. 1). Subsequently, linear fragments from the constructs containing both the 5′ and 3′ homology arms of each gene disruption segments flanking the *tuf-*aadA cassette were generated by PCR, purified by QIAquick PCR Purification Kit (Qiagen, Germantown, MD) and concentrated to 1 µg/µl in nuclease free water.

For constructing the Ech_0379 gene rescue template, we first generated the 3′ end 0.4 kb missing portion of the gene fragment downstream from the mutation site by PCR from *E*. *chaffeensis* genomic DNA (genomic coordinates are 374,462 to 374,837). The *Amtr*-mCherry (*Amtr*-mCh) DNA segments constituting the *Anapmasma marginale* transcription regulator (*Tr*) promoter and mCherry ORF were amplified using pCis mCherry-SS Himar A7 plasmid^15^. The gentamicin resistance gene coding sequence (Gent) was codon optimized to frequently found codons of *E*. *chaffeensis* (GenScript, Piscataway, NJ) (Supplementary Fig. S2). The Gent segment was then cloned downstream to *Amtr*-mCh fragment to generate *Amtr*-mCh-Gent fusion fragment. The 3′ end 0.4 kb Ech_0379 segment was then ligated at the 5′ end of the *Amtr*-mCh-Gent fragment by performing overlapping PCR experiment and the amplicons was subsequently cloned into the pHR-Ech_0379-*tuf-*aadA construct to replace the *tuf*-aadA segment with *Amtr*-mCh-Gent segment containing the additional 3′ end 0.4 kb Ech_0379 ORF segment (by Gibson Assembly method). The final plasmid construct (pHR-res-Ech_0379-*Amtr*-mCh-Gent) included the full length Ech_0379 ORF, followed by the *Amtr-*mCh-Gent and the 3′ end 1 kb genomic segment downstream from the mutation in Ech_0379 gene (Fig. 1). Linear fragments containing the entire Ech_0379 gene at the 5′ end, *Amtr*-mCh-Gent segment and the additional 3′3′ end 1 kb segment of Ech_0379 gene were then obtained by PCR from the recombinant plasmid; the DNA was purified and concentrated to 1 µg/µl.

### Purification of cell-free *E*. *chaffeensis* organisms

Five ml of *E*. *chaffeensis* cell culture from 80–90% infected confluent ISE6 cell culture flask was used to generate host cell-free *E*. *chaffeensis* organisms^15^. Briefly, the infected cell suspension was recovered by centrifugation at 15,000 g for 10 min at 4 °C; 1.5 ml of ice-cold 0.3 M sucrose solution and 100 µl volume of autoclaved rock tumbler grit (60/90 grit silicon carbide, Lortone, WA) was added to the cell pellet and votexed using a table top vortexer at maximum speed for 30 sec to release cell-free bacteria. The cell suspension was then centrifuged at 200 g for 10 min at 4 °C to pellet the host cell debris. The supernatant was carefully recovered into a 3 ml syringe and passed through a 1.6 µm filter (Whatman Ltd., Piscataway, NJ); the filtrate containing *E*. *chaffeensis* organisms were pelleted at 15,000 g for 10 min at 4 °C. The cell pellet was washed twice with 0.3 M ice-cold sucrose solution and the final pellet was resuspended in 45 µl of 0.3 M ice-cold sucrose solution and used immediately for electroporation experiments.

### Transformation of *E*. *chaffeensis* and clonal isolation of mutants

Three µg of purified linear DNA fragments from the allelic exchange mutagenesis constructs for Ech_0230 or Ech_0379 (outlined above) were added to the host cell-free *E*. *chaffeensis* organisms in 45 µl volume, mixed gently and transferred the contents into a 1 mm gap electroporation cuvette (Bio-Rad Laboratories, Hercules, CA). The cuvette was incubated on ice for 15 min and then subjected to electroporation at 2,000 volts, 25 µF and 400 Ω setting (Gene Pulser Xcell™, Bio-Rad Laboratories, Hercules, CA). The electroporated cells were transferred to a micro centrifuge tube containing 0.5 ml of stock FBS and 1 ml of uninfected ISE6 cell suspension containing about 1 × 10^6^ ISE6 cells in tick cell culture infection media. The mixed sample was centrifuged at 5,000 g for 5 min, incubated at room temperature for 15 min, cells were then resuspended in 5 ml culture media and the entire contents were transferred to a T25 flask having confluent ISE6 cells and incubated for 24 h in a humidified 34 °C incubator. After 24 h, 100 µg/ml each of spectinomycin and streptomycin were added to the culture medium; incubations were continued at 34 °C for several weeks to select clonally purified mutants. Typically, mutants were detected by PCR analysis after 2-3 weeks, although the assessment continued for several weeks beyond this time point to clear all wild type organisms.

Similar allelic exchange mutation experiment was carried out to obtain Ech_0379 gene restoration mutant from the mutant organisms having the gene mutation, except that the medium containing 80 µg/ml of gentamicin was used after 24 h of electroporation. The presence of Ech_0379 gene restoration mutant cultures was also assessed for mCherry expression using a Nikon Diaphot inverted microscope (Nikon, Melville, NY). Once identified, the antibiotic resistant host cell free infectious form (dense core cells) of the mutant cultures were used to infect DH82 cells. The mutants’ growth in DH82 cultures and similarly in ISE6 cells was maintained continuously. Further, following the establishment of clonal purity, antibiotics from the culture media were eliminated. Liquid nitrogen stocks were prepared and stored within the first two weeks after the establishment of wild-type pathogen-free mutant cultures.

### Confirming the presence of *E*. *chaffeensis* mutants

The cultures *of E*. *chaffeensis*, which grew well in the presence of antibiotics, were screened for allelic exchange mutations by genomic DNA analysis. Genomic DNAs recovered from the cultures were used to perform three different PCR assays. PCRs I and II targeted to 1) the genomic region 5′ to the allelic exchange sites and to the insertion specific DNA; 2) the insertion DNA and to the 3′ of the allelic exchange sites on the genome. PCR III was designed to test the clonal purity of mutants; primers used in this assay were targeted to the genomic regions upstream and downstream of the allelic exchange insertion sites. PCR products were resolved on a 0.9% agarose gel to identify specific predicted amplicons and then subjected to sequencing analysis to map the genomic junctions of the insertions from both ends of the amplicons. Mutations and clonal purity was further confirmed by Southern blot analysis of restriction enzyme digested DNAs; genomic DNAs from wild type and mutant organisms were subjected to restriction enzyme digestions using ClaI, EcoRI or HindIII, resolved on a 1% agarose gel and transferred to a nylon membrane (Roche Diagnostics, Indianapolis, IN)^44^. The insertion specific aadA gene segment probe was used in the blot hybridization experiment to locate inserted DNA in targeted disrupted mutants of Ech_0230 and Ech_0379. Ech_0379 gene segment probe was used for locating the insertion and restoration mutant clones of Ech_0379^47^.

### RNA analysis by RT-PCR to verify the loss and restoration of transcription

Total RNAs from wild type and mutant *E*. *chaffeensis* organisms grown in ISE6 or DH82 cell cultures were isolated by following the Tri-reagent total RNA isolation method (Sigma-Aldrich, St. Louis, MO). RNA samples were treated with RQ1 DNase (Promega, Madison, WI) to remove any residual genomic DNAs. Primers targeting to Ech_0230 or Ech_0379 ORF were used in RT-PCR analysis and the presence of specific amplicons was assessed by 1% agarose gel analysis and by subjecting the products to DNA sequence analysis^48^. Semi quantitative RT-PCR assays were performed as we previously described^48^ for assessing the mRNA expression from the genes Ech_0378, Ech_0379 and Ech_0380 using equal quantities of *E*. *chaffeensis* RNAs recovered from wild type and Ech_0379 gene disruption and restoration mutants. The assays were performed at 30, 35 and 40 cycles.

### Phenotypic characterization of Ech_0379 gene product of wild type, mutated and gene restored *E*. *chaffeensis*

Amplicons generated by PCR III using primers targeting to the Ech_0379 genomic regions upstream and downstream of the allelic exchange insertion sites of wild type, disruption mutant and restoration mutant *E*. *chaffeensis* were cloned into the EcoRV site of the plasmid, pBluescript II SK(+) by following standard molecular methods^47^. The amplicons included the entire promoter segments upstream to the Ech_0379 coding region. The *E*. *coli* NhaA gene encoding for one of the inactivated antiporter proteins along with its own promoter segment were also cloned into pBlueScript II SK(+) plasmid (Stratagene, San Diego, CA) to serve as a positive control. Primers for amplifying *E*. *coli* NhaA gene were listed in Table S1. The presence of inserts in each recombinant plasmid were verified by restriction enzyme digestion analysis and then confirmed by DNA sequencing analysis. The recombinant plasmids were then transformed into the EP432 strain of *E*. *coli* obtained from the Genetic Stock Center (New Haven, CT). This strain is a mutant for two of the three antiporter protein genes (NhaA and NhaB) and making it sensitive to growth in the presence of NaCl compared to a wild type strain^33^. It is commonly used to define the antiporter activity of bacterial antiporter genes by functional complementation assays^34,35^. To serve as a negative control, we also transformed EP432 strain with a non-recombinant pBlueScript II SK(+). Transcripts of Ech_0379 in the transformed EP432 were assessed by RT-PCR analysis using primers targeting to Ech_0379 ORF as described in the previous paragraph. Total RNAs recovered from the transformed *E*. *coli* strains were isolated by following the Tri-reagent RNA isolation method and treated with RQ1 DNase to remove any residual genomic DNAs prior to performing RT-PCR analysis.

For determining the growth complementation by antiporter genes, an isolated colony each grown in LBK medium (10 g/L tryptone, 5 g/L yeast extract, and 6.5 g/L KCl) at pH 7.0 was used to regrow in the LB media (100 mM Tris-HCl pH 7.0, 10 g/L tryptone, 5 g/L yeast extract) at pH 7.0 containing 200 mM NaCl. The LBK grown overnight cultures were diluted to 0.02 optical density (OD) at 600 nm in LB media with NaCl and then allowed to grow until 0.2 OD. At this point, 200 μl of the cultures each were transferred in triplicates wells of a 96 well microtiter plate. The plate was then incubated in a Microbiology Reader Bioscreen C (Oy Growth Curves Ab Ltd, Helsinki, Finland) at 37 °C with continuously shaking. The growth in each well was monitored by measuring OD 600 nm every 15 min for up to 11 h. All cultures were grown in triplicate wells and the average values with standard deviations were plotted as growth curves. Growth differences at 10 and 11 h of growth were compared by performing student’s *t*-test in the MS Excel software.

## Electronic supplementary material

Supplementary Figures and Tables

## References

[1] Elwell C, Mirrashidi K, Engel J (2016). *Chlamydia* cell biology and pathogenesis. Nat Rev Microbiol.

[2] Walker, D.H., Paddock, C.D. & Dumler, J.S. Emerging and re-emerging tick-transmitted rickettsial and ehrlichial infections. *Med Clin of North Am* **92**, 1345–1361, x (2008). 10.1016/j.mcna.2008.06.002 19061755

[3] Sahni SK, Narra HP, Sahni A, Walker DH (2013). Recent molecular insights into rickettsial pathogenesis and immunity. Future Microbiol.

[4] Merhej V, Raoult D (2011). Rickettsial evolution in the light of comparative genomics. Biol Rev Camb Philos Soc.

[5] Sallstrom B, Andersson SG (2005). Genome reduction in the alpha-Proteobacteria. Curr Opin Microbiol.

[6] Brayton KA, Knowles DP, McGuire TC, Palmer GH (2001). Efficient use of a small genome to generate antigenic diversity in tick-borne ehrlichial pathogens. Proc Natl Acad Sci USA.

[7] Dunning Hotopp JC (2006). Comparative genomics of emerging human ehrlichiosis agents. PLoS Genet.

[8] Nelson CM (2008). Whole genome transcription profiling of *Anaplasma phagocytophilum* in human and tick host cells by tiling array analysis. BMC Genomics.

[9] Albrecht M (2011). The transcriptional landscape of *Chlamydia pneumoniae*. Genome Biol.

[10] Renesto P (2008). *Rickettsia conorii* transcriptional response within inoculation eschar. PloS One.

[11] Lin M, Kikuchi T, Brewer HM, Norbeck AD, Rikihisa Y (2011). Global proteomic analysis of two tick-borne emerging zoonotic agents: *Anaplasma phagocytophilum* and *Ehrlichia chaffeensis*. Front Microbiol.

[12] Felsheim RF (2010). Transformation of *Anaplasma marginale*. Vet Parasitol.

[13] Felsheim RF (2006). Transformation of *Anaplasma phagocytophilum*. BMC Biotechnol.

[14] Liu ZM, Tucker AM, Driskell LO, Wood DO (2007). Mariner-based transposon mutagenesis of *Rickettsia prowazekii*. Appl Environ Microbiol.

[15] Cheng C (2013). Targeted and random mutagenesis of *Ehrlichia chaffeensis* for the identification of genes required for *in vivo* infection. PLoS Pathog.

[16] Crosby FL (2014). Knockout of an outer membrane protein operon of *Anaplasma marginale* by transposon mutagenesis. BMC Genomics.

[17] Martinez E, Cantet F, Fava L, Norville I, Bonazzi M (2014). Identification of OmpA, a *Coxiella burnetii* protein involved in host cell invasion, by multi-phenotypic high-content screening. PLoS Pathog.

[18] Driskell LO (2009). Directed mutagenesis of the *Rickettsia prowazekii pld* gene encoding phospholipase D. Infect Immun.

[19] Binet R, Maurelli AT (2009). Transformation and isolation of allelic exchange mutants of *Chlamydia psittaci* using recombinant DNA introduced by electroporation. Proc Natl Acad Sci USA.

[20] Mueller KE, Wolf K, Fields KA (2016). Gene Deletion by Fluorescence-Reported Allelic Exchange Mutagenesis in *Chlamydia trachomatis*. MBio.

[21] Noriea, N. F., Clark, T. R. & Hackstadt, T. Targeted knockout of the *Rickettsia rickettsii* OmpA surface antigen does not diminish virulence in a mammalian model system. *MBio* **6** (2015). 10.1128/mBio.00323-15 PMC4453529 25827414

[22] Wood DO, Wood RR, Tucker AM (2014). Genetic systems for studying obligate intracellular pathogens: an update. Curr Opin Microbiol.

[23] McClure, E. E. *et al*. Engineering of Obligate Intracellular Bacteria: Progress, Challenges, and Paradigms. *Nat Rev Microbiol*, 10.1038/nrmicro.2017.59 (2017). 10.1038/nrmicro.2017.59 PMC5557331 28626230

[24] Yabsley MJ (2010). Natural history of *Ehrlichia chaffeensis*: vertebrate hosts and tick vectors from the United States and evidence for endemic transmission in other countries. Vet Parasitol.

[25] Rikihisa Y (2010). *Anaplasma phagocytophilum* and *Ehrlichia chaffeensis*: subversive manipulators of host cells. Nat Rev Microbiol.

[26] Walker DH, Dumler JS (1996). Emergence of the ehrlichioses as human health problems. Emerg Infect Dis.

[27] Davidson WR (2001). Persistent *Ehrlichia chaffeensis* infection in white-tailed deer. J Wildl Dis.

[28] Unver A, Rikihisa Y, Stich RW, Ohashi N, Felek S (2002). The omp-1 major outer membrane multigene family of *Ehrlichia chaffeensis* is differentially expressed in canine and tick hosts. Infect Immun.

[29] Dumler JS, Sutker WL, Walker DH (1993). Persistent infection with *Ehrlichia chaffeensis*. Clin Infect Dis.

[30] McGill JL (2016). Vaccination with an Attenuated Mutant of *Ehrlichia chaffeensis* Induces Pathogen-Specific CD4+ T Cell Immunity and Protection from Tick-Transmitted Wild-Type Challenge in the Canine Host. PloS One.

[31] Nair AD (2015). Attenuated Mutants of *Ehrlichia chaffeensis* Induce Protection against Wild-Type Infection Challenge in the Reservoir Host and in an Incidental Host. Infect Immun.

[32] Yamamoto H (2014). EF-G and EF4: translocation and back-translocation on the bacterial ribosome. Nat Rev Microbiol.

[33] Pinner E, Kotler Y, Padan E, Schuldiner S (1993). Physiological role of NhaB, a specific Na^+/^H^+^ antiporter in *Escherichia coli*. J Biol Chem.

[34] Lentes CJ (2014). Molecular characterization of the Na^+/^H^+^-antiporter NhaA from *Salmonella Typhimurium*. PloS One.

[35] Dzioba-Winogrodzki J (2009). The *Vibrio cholerae* Mrp system: cation/proton antiport properties and enhancement of bile salt resistance in a heterologous host. J Mol Microbiol Biotechnol.

[36] Hyde JA (2011). Bioluminescent imaging of *Borrelia burgdorferi in vivo* demonstrates that the fibronectin-binding protein BBK32 is required for optimal infectivity. Mol Microbiol.

[37] Crosby FL (2015). Reduced Infectivity in cattle for an outer membrane protein mutant of *Anaplasma marginale*. Appl Environ Microbiol.

[38] Oliva Chavez AS (2015). An O-Methyltransferase Is Required for Infection of Tick Cells by *Anaplasma phagocytophilum*. PLoS Pathog.

[39] Falkow S (2004). Molecular Koch’s postulates applied to bacterial pathogenicity [mdash] a personal recollection 15 years later. Nat Rev Microbiol.

[40] Campbell-Valois FX, Sansonetti PJ (2014). Tracking bacterial pathogens with genetically-encoded reporters. FEBS Letters.

[41] Lin M (2016). *Ehrlichia* secretes Etf-1 to induce autophagy and capture nutrients for its growth through RAB5 and class III phosphatidylinositol 3-kinase. Autophagy.

[42] Lina, T. T., Dunphy, P. S., Luo, T. & McBride, J. W. *Ehrlichia chaffeensis* TRP120 Activates Canonical Notch Signaling To Downregulate TLR2/4 Expression and Promote Intracellular Survival. *MBio* **7** (2016). 10.1128/mBio.00672-16 PMC4958247 27381289

[43] Huang B (2010). *Anaplasma phagocytophilum* APH_0032 is expressed late during infection and localizes to the pathogen-occupied vacuolar membrane. Microbial pathog.

[44] Rennoll-Bankert KE, Garcia-Garcia JC, Sinclair SH, Dumler JS (2015). Chromatin-bound bacterial effector ankyrin A recruits histone deacetylase 1 and modifies host gene expression. Cell Microbiol.

[45] Du Toit A (2016). Bacterial pathogenesis: Bacterial effectors skip a few steps. Nat Rev Microbiol.

[46] Munderloh UG (1999). Invasion and intracellular development of the human granulocytic ehrlichiosis agent in tick cell culture. J Clin Microbiol.

[47] Sambrook, J. & Russell, D. W. *Molecular Cloning: ALaboratory Manual*, *Edn*, *III*. (Cold Spring HarborLaboratory, Cold Spring Harbor, New York) (2001).

[48] Cheng C, Nair AD, Jaworski DC, Ganta RR (2015). Mutations in *Ehrlichia chaffeensis* Causing Polar Effects in Gene Expression and Differential Host Specificities. PloS One.

