# Supplementary material for: A genetic system for targeted mutations to disrupt and restore genes in the obligate bacterium, Ehrlichia chaffeensis
Source: Sci Rep. 2017 Nov 17;7:15801. doi: 10.1038/s41598-017-16023-y (PMC5693922; doi:10.1038/s41598-017-16023-y)
Supplement: Supplementary file 1 — Supplementary Figures and Tables [file 41598_2017_16023_MOESM1_ESM.pdf]

A genetic system for targeted mutations to disrupt and restore genes in the obligate bacterium, *Ehrlichia chaffeensis*

Ying Wang, Lanjing Wei, Huitao Liu, Chuanmin Cheng[#], and Roman R Ganta[*]

Center of Excellence for Vector-Borne Diseases (CEVBD), Department of Diagnostic Medicine/Pathobiology, College of Veterinary Medicine, Kansas State University, Manhattan, KS 66506, USA

[#]Present address: Vanderbilt Technologies for Advanced Genomics, Vanderbilt University Medical Center, Nashville, TN 37232

[*]To whom correspondence should be addressed. E-mail: rganta@vet.k-state.edu

Supplemental Figure 1. An illustration outlining the detailed schematic representation of the strategies employed in creating targeted allelic exchange mutations in *E. chaffeensis* to inactivate the genes Ech_0230 and Ech_0379 and to restore the inactivated gene of Ech_0379. (A and A′ refer to 5′ and 3′ homology arms.)

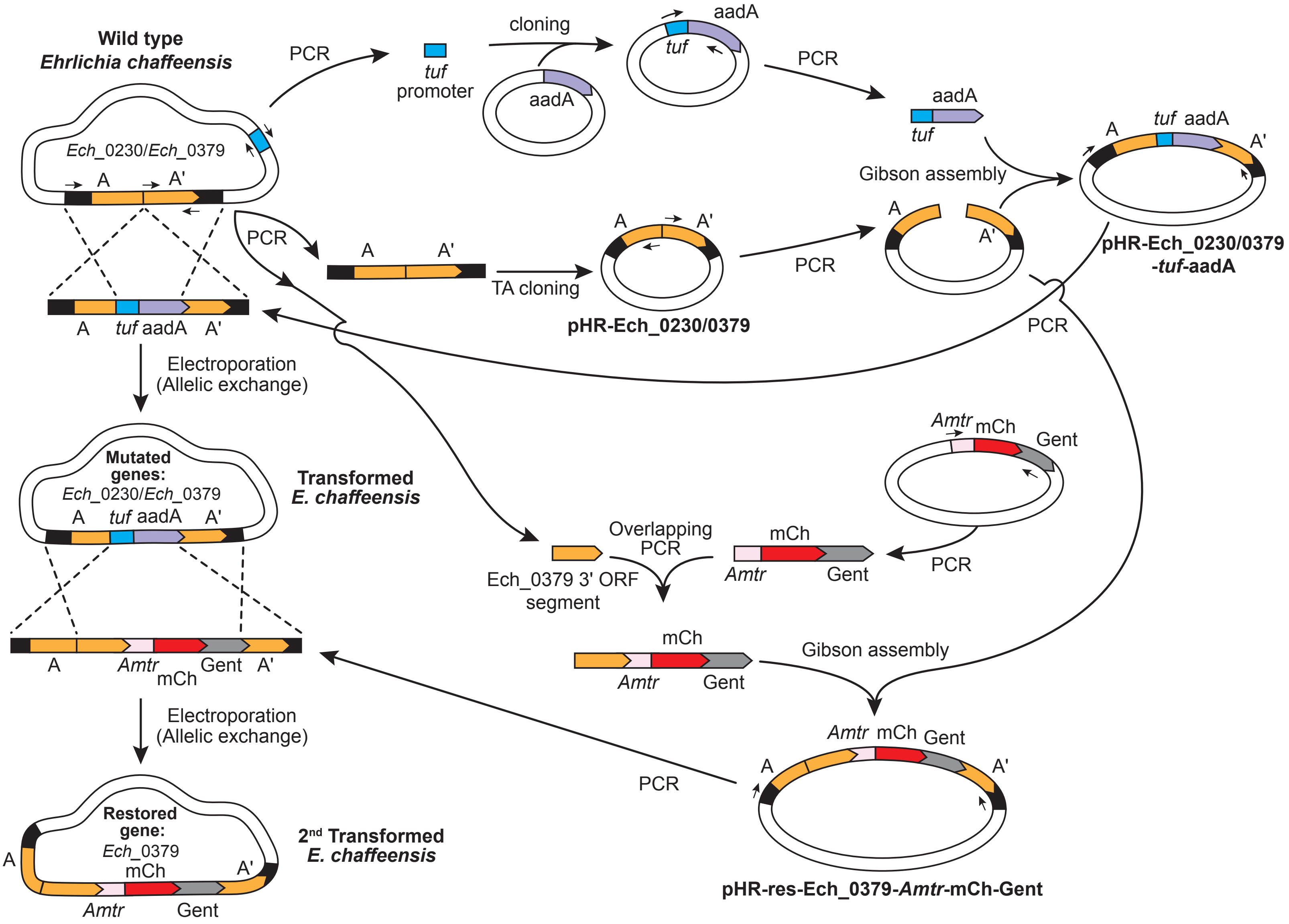

Supplemental Figure 2. Gentamicin resistance gene protein coding sequence (Gent) codon optimized for *E. chaffeensis.* E. chaffeensis genome based codon optimized sequence is shown in the upper row and the original non-optimized coding sequence and the translated protein sequence are presented in the middle and bottom rows, respectively. The sequence data were deposited in the GenBank (Accession # KY977452).

```
Optimized  1    ATGTTAAGATCATCAAATGATGTTACACAACAAGGAAGTAGACCAAAAACTAAA
Original   1    ATGTTACGCAGCAGCAACGATGTTACGCAGCAGGGCAGTCGCCCTAAAACAAAG
Protein    1    M  L  R  S  S  N  D  V  T  Q  Q  G  S  R  P  K  T  K

Optimized  55   TTAGGTGGTAGTAGTATGGGAATTATTAGAACTTGTAGATTAGGTCCTGATCAA
Original   55   TTAGGTGGCTCAAGTATGGGCATCATTCGCACATGTAGGCTCGGCCCTGACCAA
Protein    19   L  G  G  S  S  M  G  I  I  R  T  C  R  L  G  P  D  Q

Optimized  109  GTAAAAAGTATGAGAGCAGCTTTAGATTTATTTGGTAGAGAATTTGGAGATGTT
Original   109  GTCAAATCCATGCGGGCTGCTCTTGATCTTTTCGGTCGTGAGTTCGGAGACGTA
Protein    37   V  K  S  M  R  A  A  L  D  L  F  G  R  E  F  G  D  V

Optimized  163  GCAACATATTCTCAACATCAACCAGATAGTGATTATTTGGGTAATTTATTAAGA
Original   163  GCCACCTACTCCCAACATCAGCCGGACTCCGATTACCTCGGGAACTTGCTCCGT
Protein    55   A  T  Y  S  Q  H  Q  P  D  S  D  Y  L  G  N  L  L  R

Optimized  217  TCAAAAACTTTTATAGCTTTAGCAGCTTTTGATCAAGAAGCAGTAGTTGGAGCT
Original   217  AGTAAGACATTCATCGCGCTTGCTGCCTTCGACCAAGAAGCGGTTGTTGGCGCT
Protein    73   S  K  T  F  I  A  L  A  A  F  D  Q  E  A  V  V  G  A

Optimized  271  TTAGCAGCTTATGTATTACCTAGATTTGAACAACCAAGATCAGAAATTTATATA
Original   271  CTCGCGGCTTACGTTCTGCCCAGGTTTGAGCAGCCGCGTAGTGAGATCTATATC
Protein    91   L  A  A  Y  V  L  P  R  F  E  Q  P  R  S  E  I  Y  I

Optimized  325  TATGATTTAGCAGTTTCTGGTGAACATAGAAGACAAGGAATAGCAACAGCTTTA
Original   325  TATGATCTCGCAGTCTCCGGCGAGCACCGGAGGCAGGGCATTGCCACCGCGCTC
Protein    109  Y  D  L  A  V  S  G  E  H  R  R  Q  G  I  A  T  A  L

Optimized  379  ATAAATTTATTAAAACATGAAGCAAATGCTTTAGGTGCATATGTAATTTATGTT
Original   379  ATCAATCTCCTCAAGCATGAGGCCAACGCGCTTGGTGCTTATGTGATCTACGTG
Protein    127  I  N  L  L  K  H  E  A  N  A  L  G  A  Y  V  I  Y  V

Optimized  433  CAAGCTGATTATGGAGATGATCCTGCAGTAGCTTTATATACTAAATTAGGTATT
Original   433  CAAGCAGATTACGGTGACGATCCCGCAGTGGCTCTCTATACAAAGTTGGGCATA
Protein    145  Q  A  D  Y  G  D  D  P  A  V  A  L  Y  T  K  L  G  I

Optimized  487  AGAGAAGAAGTTATGCATTTTGATATTGATCCAAGTACAGCAACATAA
Original   487  CGGGAAGAAGTGATGCACTTTGATATCGACCCAAGTACCGCCACCTAA
Protein    163  R  E  E  V  M  H  F  D  I  D  P  S  T  A  T  *
```

Supplementary Table S1: List of oligonucleotides used in this study.

Primer*	Sequence (From 5' end to 3' end)**	Orientation	Size (bp)
**HOMOLOGOUS RECOMBINATION CONSTRUCTS:**			
**For Ech_0230 gene disruption**			
Ech_0230 gene segment cloning			
RRG1591	TATGGGCCTAAGATAGTATTACC	forward	2074
RRG1602	AAGACACACAAGAACATGACACTGCC	reverse	
Insertion specific primers to split the plasmid construct of pHR-Ech_0230			
RRG1599	gatcaccaaggtagtcggcaaataactcgagTATATATAATCATGTATCGATTATATATAACTGTGTGC	forward	6081
RRG1592	cctaattaaaaaaagtcaaaattaatagtcacatttttctcgagCTGTAGTACCATGTGTTACTTACCCTCTTTC	reverse	
**For Ech_0379 gene disruption**			
Ech_0379 gene segment cloning			
RRG1603	ACCTGCTGTACTGAGTATGTTCTTG	forward	2546
RRG1608	AGACAAGAACATGCTTCAGGTGCTAC	reverse	
Insertion specific primers to split the plasmid construct of pHR-Ech_0379			
RRG1604	cctaattaaaaaaagtcaaaattaatagtcacatttttctcgagTGCTGCATTAATTCTATGTAATTATCTTTAG	forward	6552
RRG1605	gatcaccaaggtagtcggcaaataactcgagTATTATGCTTTATAAATGTTCTCAGTCTATTGGC	reverse	
**For cloning *Tuf-2* (Ech_0407) promoter**			
RRG1595	AAAAATGTGACTATTAATTTTGACTTTTTTTAATTAGG	forward	387
RRG1596	gcgatcaccgcttccctcatAAACAAATACCTTTAACATCATTAAACCATTTC	reverse	
**For cloning aadA gene**			
RRG1597	gaaatggtttaatgatgttaaaggtatttgtttATGAGGGAAGCGGTGATCGC	forward	789
RRG1598	TTATTTGCCGACTACCTTGGTGATC	reverse	
**For Ech_0379 gene function restoration**			
Ech_0379 3' end segment cloning			
RG8	GATAATTACATAGAATTAATGCAGCATATTATGCTTTATAAATGTTCTCAG	forward	427
RG9	GCATGCGGCGATCGTTCTAGGAGCTATAAATCTACACTTTCTTCAAC	reverse	
For cloning Amtr promoter with mCherry gene (Amtr-mCh)			
RG10	CTCCTAGAACGATCGCCGCATGCTAGC	forward	950
RG11	AATTTAATCCCTATTTGTATAATTCG	reverse	
For cloning gentamycin gene from plasmid pEch_rpsI-GENT			
RG12	atacaaatagggattaaattATGTTAAGATCATCAAATGATG	forward	563

RRG914	ACTACTAGTTTATGTTGCTGTACTTGGATCAATATC	reverse	

Insertion specific primers to split the plasmid construct of pHR-Ech_0379 for rescue construct

RG6	TGCTGCATTAATTCTATGTAATTATCTTTAG	forward	6499
RG22	tacagcaacataaactagtagtTATTATGCTTTATAAATGTTCTCAGTCTATTGGC	reverse	

PRIMERS FOR MUTANT SCREENING:

Ech_0230 disruption mutant

PCR I

RRG1944	ATTAGTGCTATGGCATTTGGTC	forward	1525
RRG1596		reverse	

PCR II

RRG1597		forward	2057
RRG1945	CAATTTACATGACATACTAACAAGC	reverse	

PCR III

RRG1944		forward	3582
RRG1945		reverse	

Ech_0379 disruption mutant

PCR I

RRG1946	TGAGTGCTATGATACTCAAAGC	forward	1779
RRG1596		reverse	

PCR II

RRG1597		forward	2352
RRG1947	AGAATCAACAAGGCCTACATACC	reverse	

PCR III

RRG1946		forward	4131
RRG1947		reverse	

Ech_0379 rescue mutant

PCR I

RRG1946		forward	2243
RG97	TCCGCAGGATGTTTCACATA	reverse	

PCR II

RRG94	AAGCAAATGCTTTAGGTGCAT	forward	1711
RRG1947		reverse	

PCR III			
RRG1946		forward	4824
RRG1947		reverse	
**<u>SOUTHERN BLOT PROBE AMPLIFICATION PRIMERS:</u>**			
**<u>aadA gene probe</u>**			
RRG1200	GTTACGGTGACCGTAAGGCTT	forward	603
RRG1201	CACGTAGTGAACAAATTCTTCCAACTG	reverse	
**<u>Ech_0379 gene probe</u>**			
RRG1282	TGAAAATCTGATCGATAGTGCTGTGG	forward	384
RRG1283	GGTTGCATTCCCTACAACCTTAG	reverse	
**<u>RT-PCR PRIMERS:</u>**			
**<u>Ech_0230</u>**			
RG26	GCTTTGGATTGTTTGTCTTA	forward	320
RG27	TCCATCCCATAACAAATCTA	reverse	
**<u>Ech_0379</u>**			
RRG1276	CTAAGGTTGTAGGGAATGCAACC	forward	376
RRG1277	ACAAGGTAAGTACCTTGCTTGCTC	reverse	
**<u>Ech_0378</u>**			
RRG1632	TGCTATAGGGATACCTGTAGCTTTTGC	forward	447
RRG1633	GCAAGACCATCGTACGTACTAGGTG	reverse	
**<u>Ech_0380</u>**			
RRG1634	ATGTGCTCTGTATCAATTGCTTG	forward	182
RRG1635	AACAAAGAAGTAAAAAGACATACATG	reverse	
**FOR CLONING NhaA GENE WITH ITS PROMOTER:**			
RRG2158	GCTCATTTCTCTCCCTGATAACA	forward	1817
RRG2159	TGCTCTCTTCTCCTTGACCTTAC	reverse	

*Sequence for the primers was provided only once if a primer is listed multiple times

**Uppercase sequences are gene specific; lowercase sequences are Gibson Assembly overlaps added during primer designing

Supplementary Table S2: Plasmids used and constructed in this study

Name	Description	Reference
pHR-Ech_0230	Ech_0230 homology arms; pCR™2.1-TOPO vector	This study
pHR-Ech_0379	Ech_0379 homology arms, pCR™2.1-TOPO vector	This study
pHR-Ech_0230-*tuf*-aadA	Ech_0230 homology arms, aadA expression driven by *tuf-2* promoter, pCR™2.1-TOPO vector	This study
pHR-Ech_0379-*tuf*-aadA	Ech_0379 homology arms, aadA expression driven by *tuf-2* promoter, pCR™2.1-TOPO vector	This study
pHR-res-Ech_0379-*Amtr*-mCh-Gent	Ech_0379 homology arms, mCherry and gentamycin expression driven by *Amtr* promoter, pCR™2.1-TOPO vector	This study
pEch_*rpsl*-GENT	Codon optimized gentamicin resistant gene for E. chaffeensis genome, PUC57 vector	This study

A genetic system for targeted mutations to disrupt and restore genes in the obligate bacterium, *Ehrlichia chaffeensis*

Ying Wang, Lanjing Wei, Huitao Liu, Chuanmin Cheng, and Roman R Ganta

---

Raw data used in generating Figures 2, 3, 4, 5 and 6:

Figure 2B (PCR I) and (PCR II): Note, markers lane (L) is the same for both and the first set of W and M are for PCR I and second set of W and M are for PCR II.

L W M W M

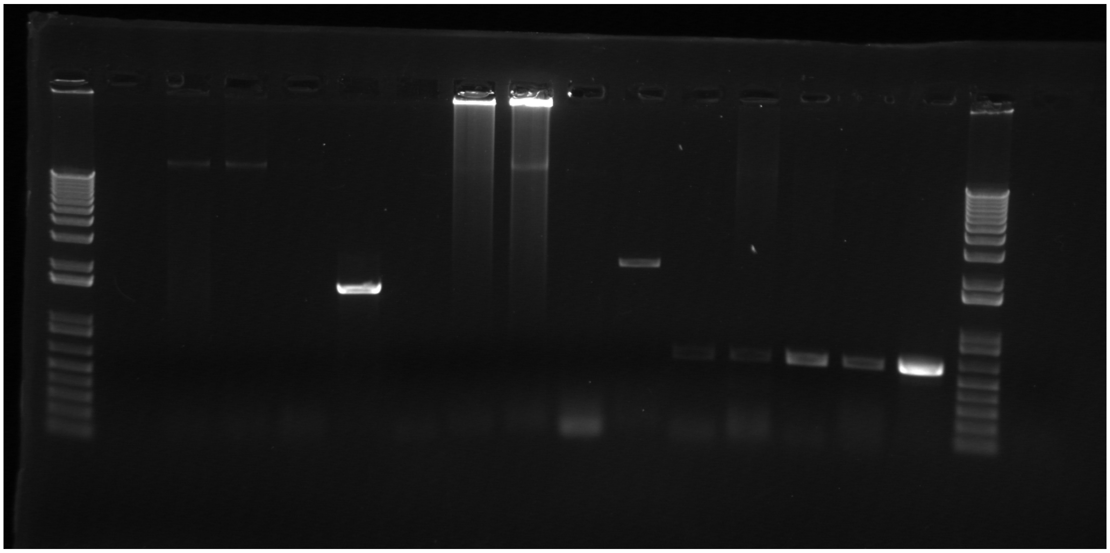

Figure 2B (PCR III):

L W M -

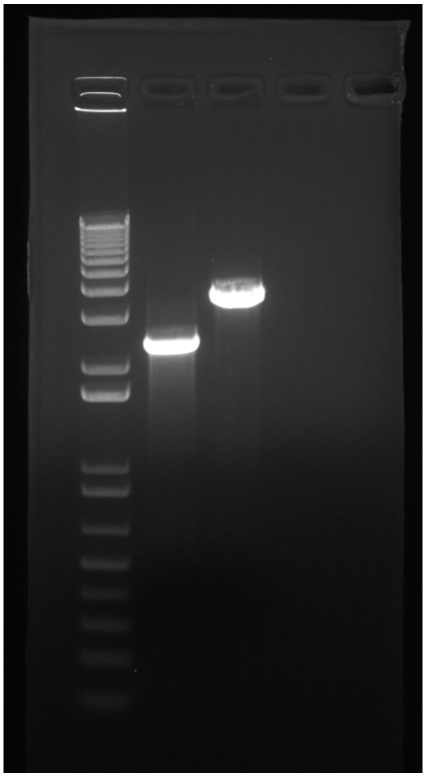

Figure 2D S-blot:

W M

C C E kb

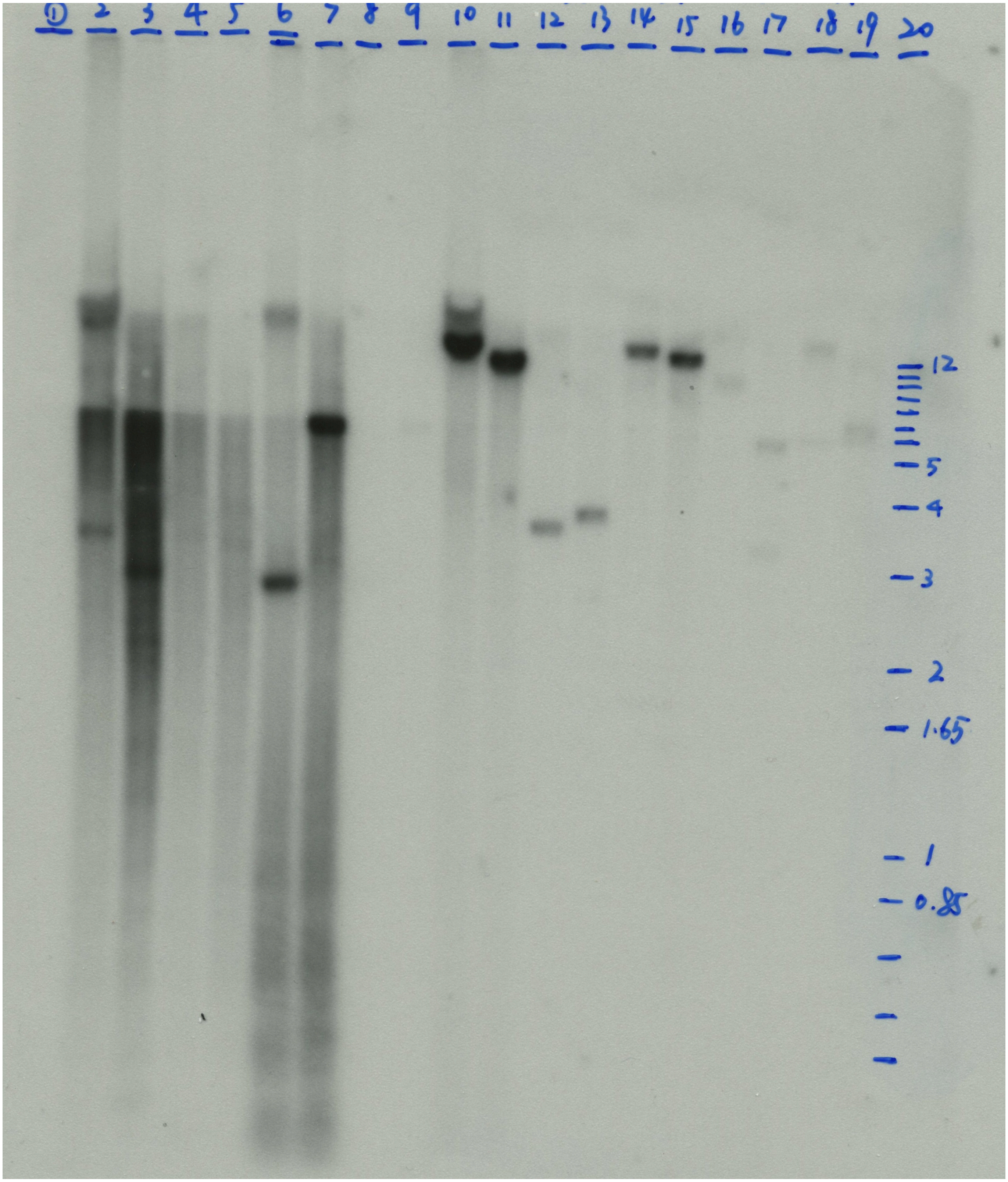

Figure 3B (PCR I) and (PCR II): Note, markers lane (L) is the same for both and the first set of W and M are for PCR I and second set of W and M are for PCR II.

L W M W M

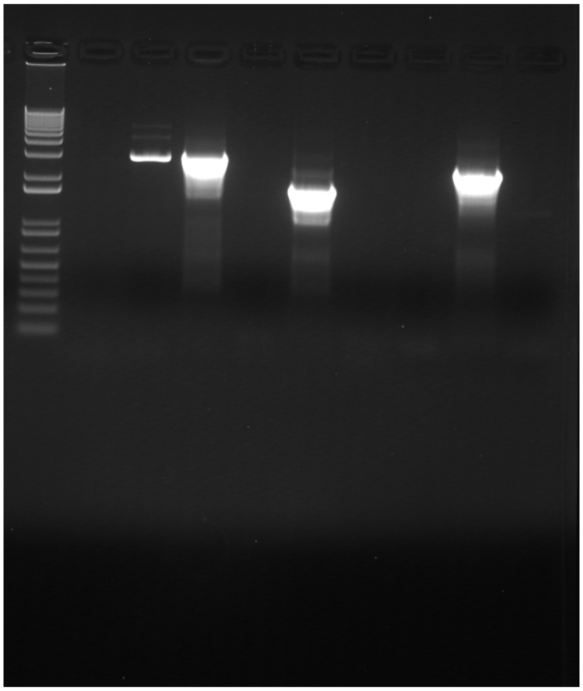

Figure 3B (PCR III):

L W M -

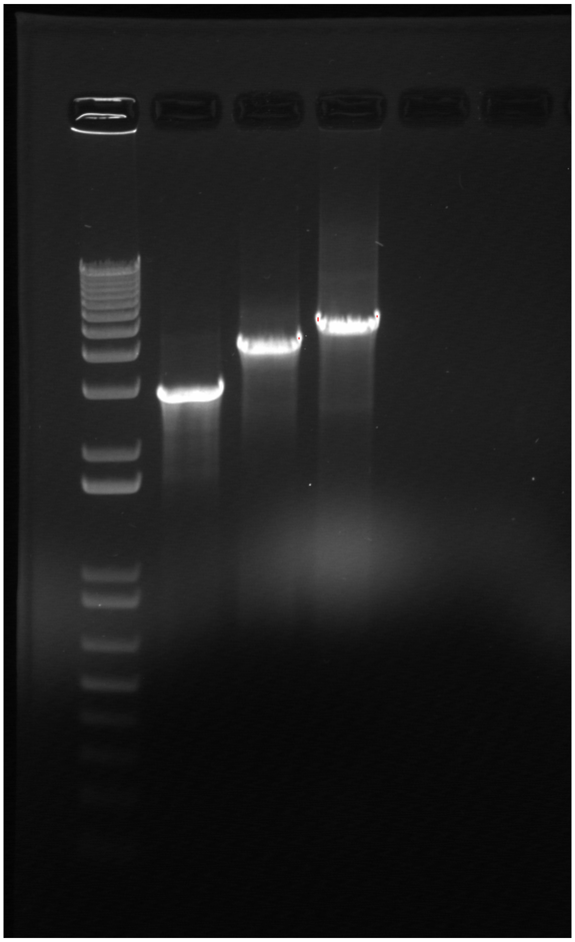

Figure 3D S-blot:

W M

C C H kb

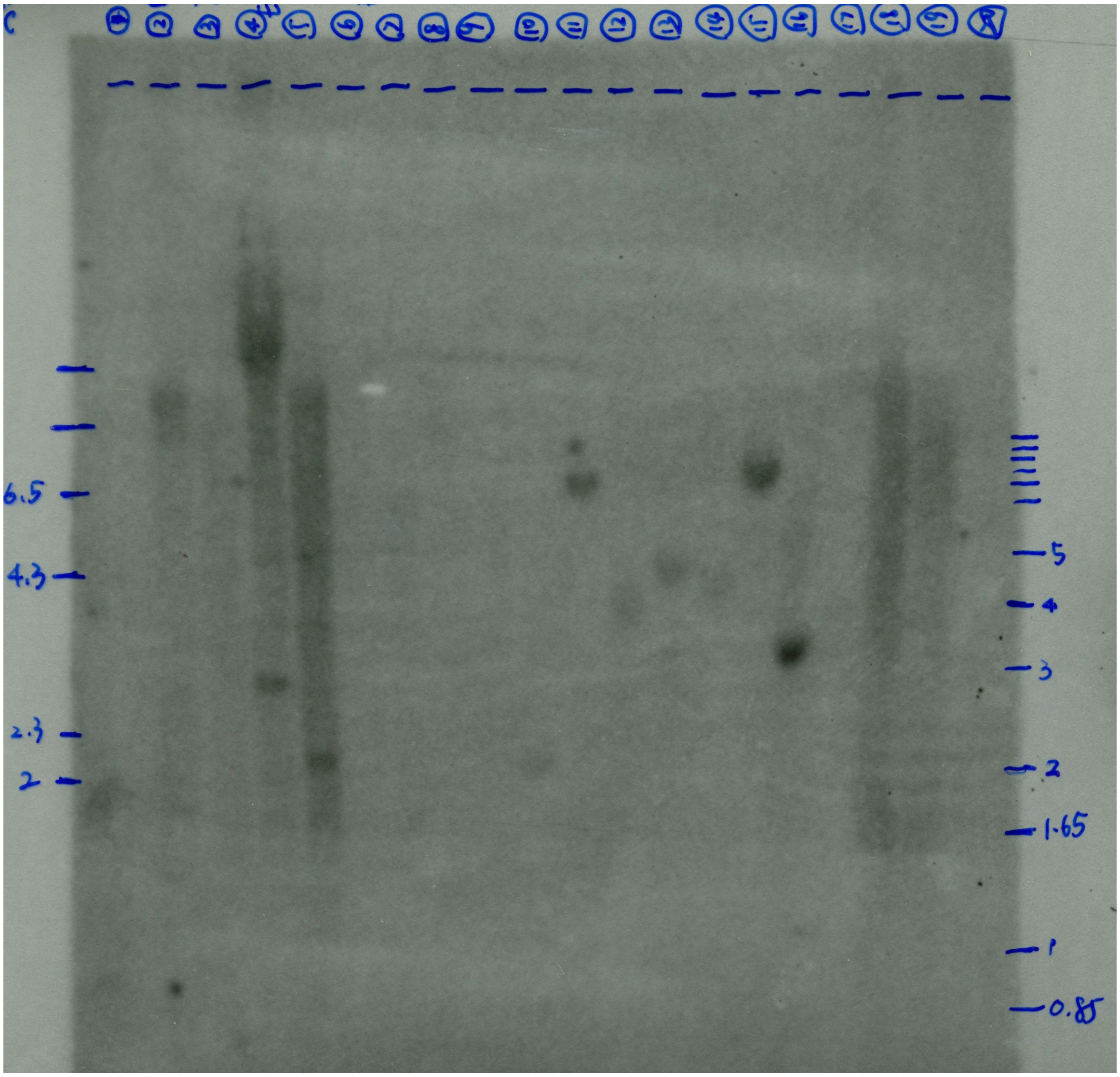

<u>Figure 4C (PCR I) and (PCR II)</u>: Note, markers lane (L) is the same for both and the first set of W and M are for PCR I and second set of W and M are for PCR II.

L W R L W R

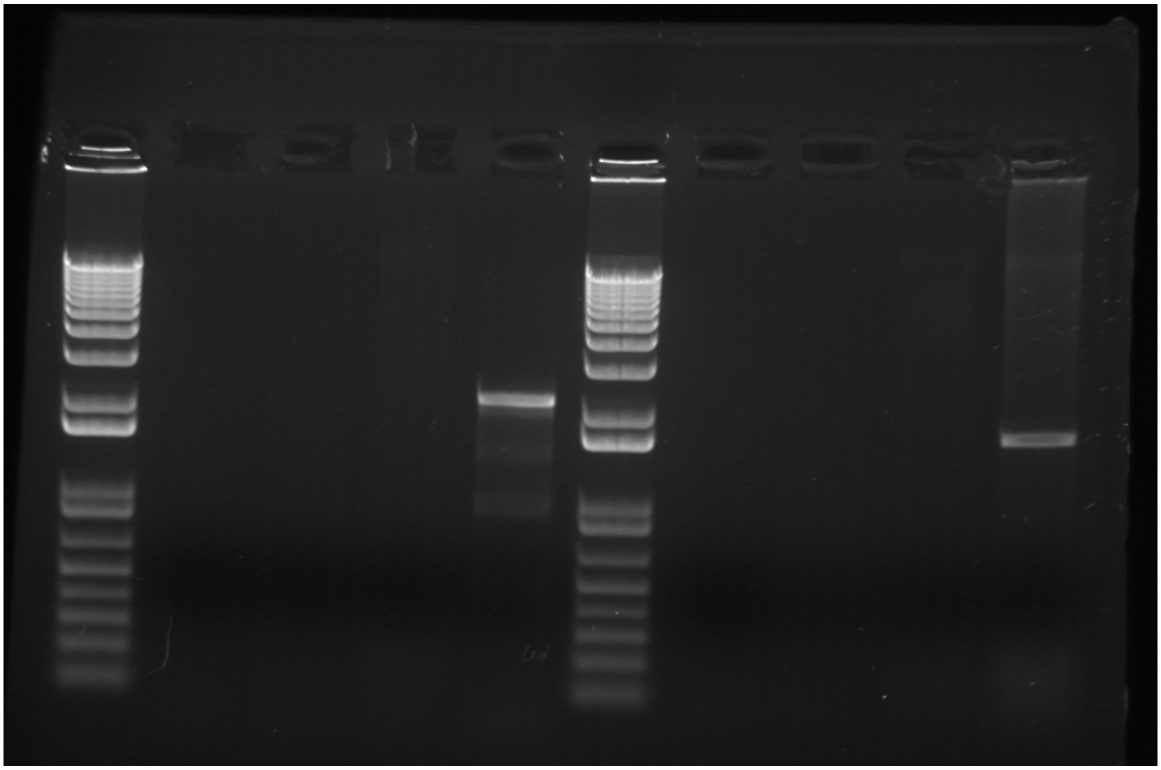

<u>Figure 4C (PCR III)</u>:

L M R -

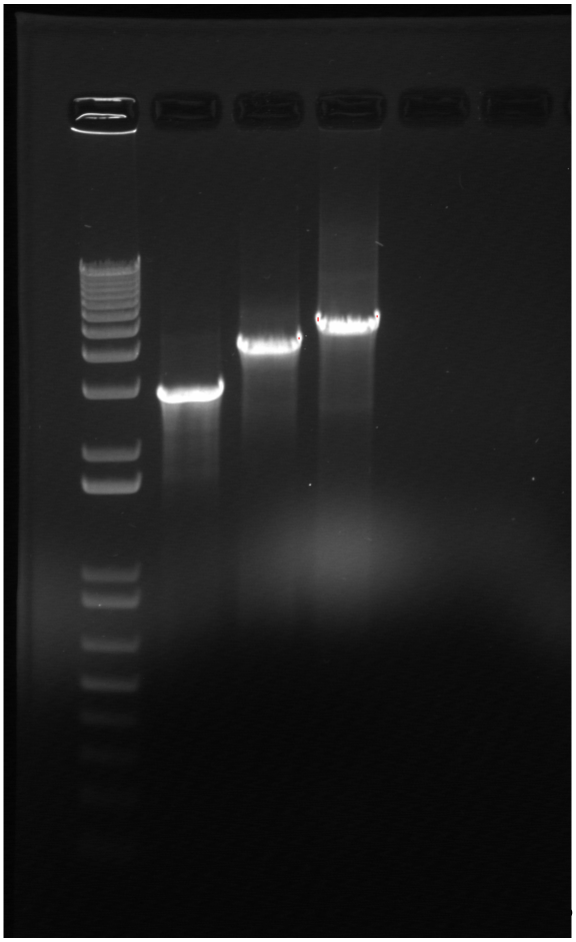

Figure 4E S-blot:

W M$_1$ M$_2$ R$_1$ R$_2$ kb

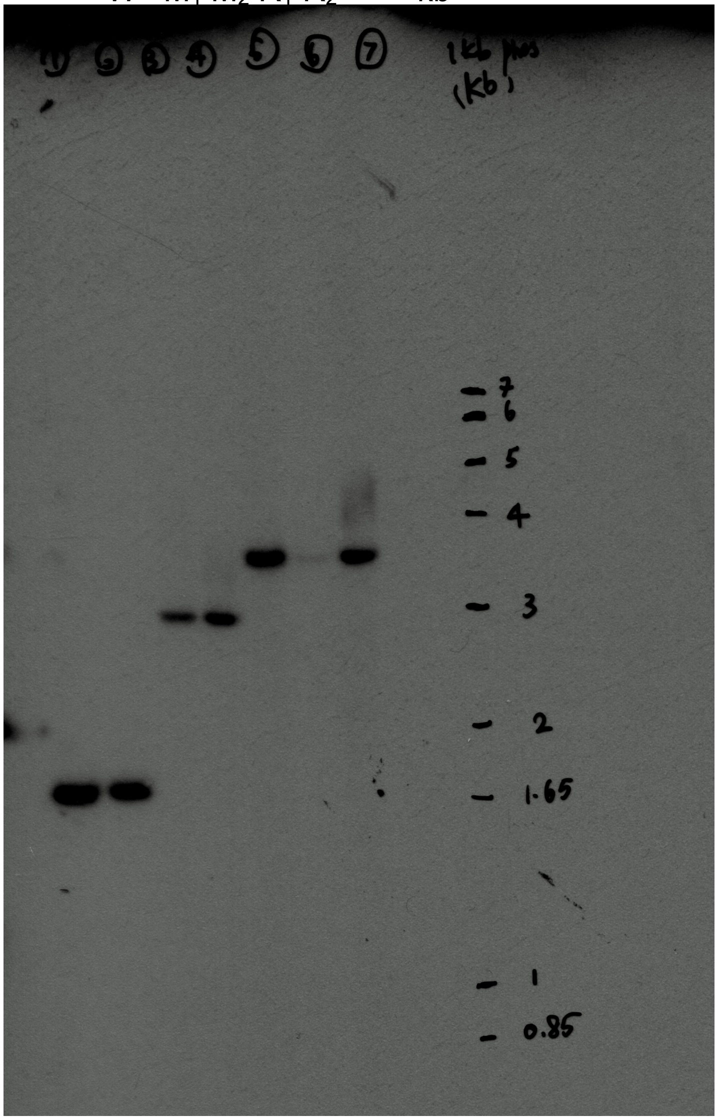

Figure 5A (RT-PCR):

L W M + -

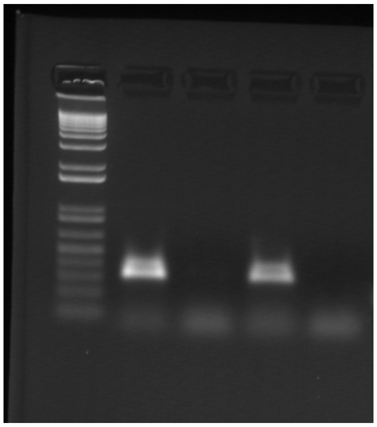

Figure 5B (RT-PCR):

L W M R W M R -

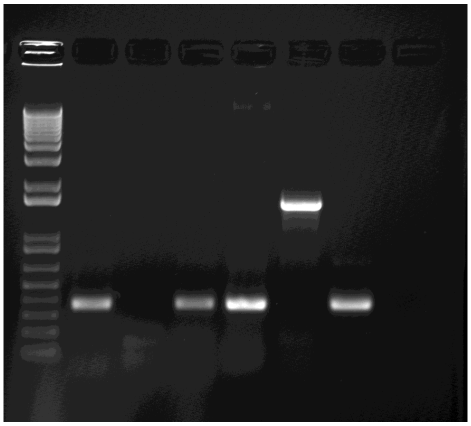

Figure 5C (Ech_0378 and Ech_0380, respectively):

L W M R + - L W M R + -

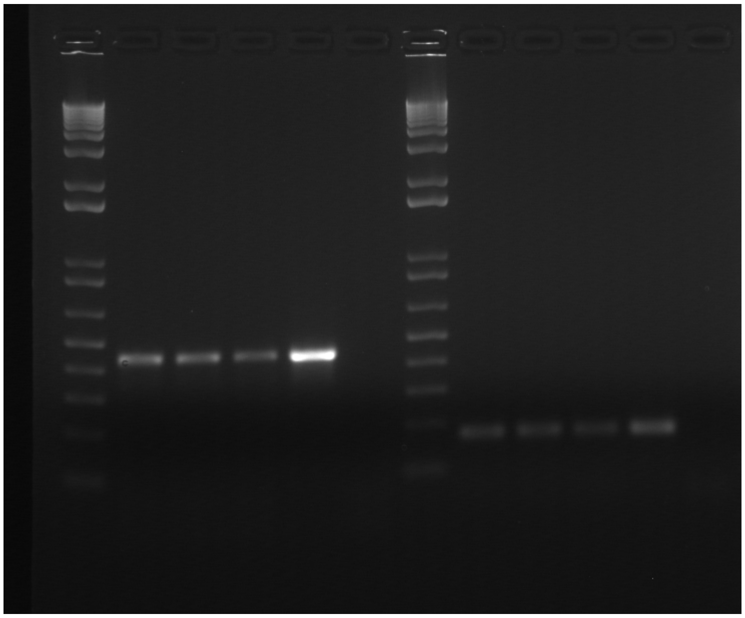

Figure 5C (Ech_0379):

L W M R + -

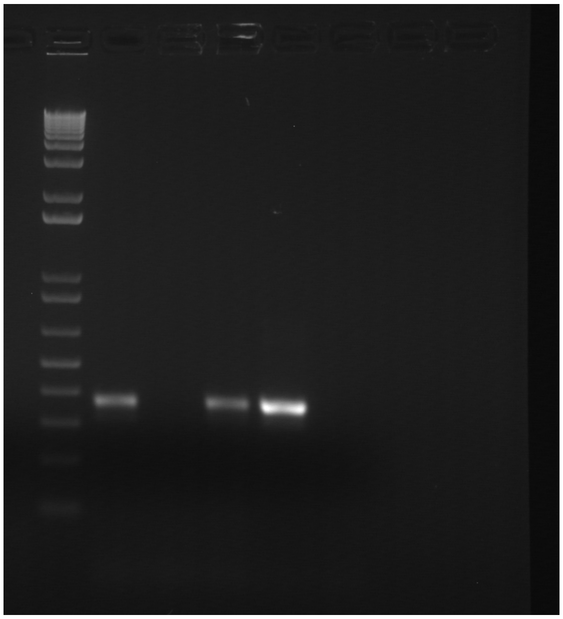

<u>Raw data used in generating Figure 6B:</u>

		E. coli NhaA			pBSK			0379 wild (LBK)			0379 mutant (LBK)			0379 rescued	
0	0.183241	0.181241	0.181241	0.236241	0.233241	0.233241	0.228241	0.226241	0.225241	0.209241	0.209241	0.208241	0.209241	0.203241	0.200241
1	0.355241	0.360241	0.369241	0.386241	0.388241	0.393241	0.431241	0.429241	0.433241	0.377241	0.377241	0.376241	0.373241	0.365241	0.360241
2	0.458241	0.473241	0.496241	0.473241	0.476241	0.483241	0.548241	0.545241	0.552241	0.489241	0.488241	0.487241	0.493241	0.485241	0.482241
3	0.507241	0.522241	0.542241	0.486241	0.489241	0.496241	0.575241	0.571241	0.580241	0.526241	0.522241	0.530241	0.552241	0.542241	0.543241
4	0.524241	0.539241	0.557241	0.490241	0.494241	0.502241	0.591241	0.587241	0.597241	0.529241	0.528241	0.532241	0.566241	0.557241	0.558241
5	0.534241	0.548241	0.565241	0.488241	0.493241	0.502241	0.596241	0.591241	0.602241	0.523241	0.525241	0.522241	0.566241	0.554241	0.554241
6	0.537241	0.550241	0.566241	0.485241	0.491241	0.499241	0.592241	0.588241	0.601241	0.517241	0.513241	0.516241	0.576241	0.566241	0.566241
7	0.536241	0.549241	0.563241	0.480241	0.486241	0.494241	0.590241	0.584241	0.598241	0.513241	0.510241	0.511241	0.588241	0.578241	0.578241
8	0.533241	0.545241	0.560241	0.475241	0.481241	0.488241	0.588241	0.582241	0.599241	0.505241	0.501241	0.503241	0.595241	0.581241	0.578241
9	0.527241	0.539241	0.552241	0.468241	0.473241	0.482241	0.583241	0.578241	0.594241	0.499241	0.495241	0.497241	0.593241	0.580241	0.579241
10	0.521241	0.533241	0.547241	0.462241	0.469241	0.476241	0.577241	0.572241	0.587241	0.490241	0.488241	0.488241	0.595241	0.581241	0.577241
11	0.515241	0.526241	0.539241	0.456241	0.461241	0.468241	0.571241	0.564241	0.580241	0.484241	0.483241	0.482241	0.586241	0.570241	0.568241
12	0.514241	0.523241	0.534241	0.450241	0.453241	0.459241	0.565241	0.559241	0.574241	0.476241	0.473241	0.471241	0.577241	0.563241	0.559241
13	0.503241	0.513241	0.523241	0.441241	0.444241	0.449241	0.553241	0.548241	0.564241	0.466241	0.466241	0.464241	0.569241	0.554241	0.549241
